# Dynamic transcriptional immune landscape in response to NK-cell therapy combined with gemcitabine plus S-1 in advanced pancreatic cancer: a phase 1b/2 trial

**DOI:** 10.1038/s41392-025-02488-1

**Published:** 2025-11-21

**Authors:** Qin Tan, Yifei Li, Caixia Liu, Jing Xu, Jinlian Tong, Jiangyong Yu, Yingying Huang, Xueqing Hu, Sen Qin, Fei Xiao, Yunbo Zhao, Jie Ma

**Affiliations:** 1 Center of Biotherapy, Beijing Hospital, National Center of Gerontology, Institute of Geriatric Medicine, Chinese Academy of Medical Science, Beijing, 100730 China; 2 Clinical Biobank, Beijing Hospital, National Center of Gerontology, National Health Commission, Institute of Geriatric Medicine, Chinese Academy of Medical Science, Beijing, 100730 China; 3 Department of Oncology, Beijing Hospital, National Center of Gerontology, Institute of Geriatric Medicine, Chinese Academy of Medical Science, Beijing, 100730 China

**Keywords:** Gastrointestinal cancer, Cancer therapy, Immunotherapy, Gastrointestinal cancer

## Abstract

Despite advancements in several malignancies, the treatment atlas of natural killer (NK) cell therapy for pancreatic cancer remains inadequate, and the dynamic immune landscape underlying the various responses is still incompletely understood. This phase 1b/2 trial evaluated the safety and efficacy of allogeneic NK cell therapy combined with gemcitabine and S-1 as a first-line treatment for advanced pancreatic cancer (APC) and explored the dynamic responsive immune landscape (ChiCTR1900021764). The administration of 1 × 10^9^ to 8 × 10^9^ NK cells to 24 patients was well tolerated, with no graft-versus-host disease or dose-limiting toxicity. Among the 19 evaluable patients, the objective response rate was 31.6%, and the disease control rate was 73.7%. The median progression-free survival was 6.6 months, and the overall survival was 10.8 months. Further longitudinal single-cell RNA sequencing (scRNA-seq) of 19 paired-blood samples revealed an increased proportion of certain NK cell subsets (c4-ZEB2, c5-IL7, c6-IL15, c10-NCR3, and c11-TNFSF8) and T-cell subsets (CD8^+^ Teff and CD4^+^ Tem) in responders, characterized by increased expression of proinflammatory and effector molecules. Bulk T-cell receptor (TCR) Vβ repertoire sequencing of responders indicated potential T-cell clonal expansion, manifested as a greater abundance of large and hyperexpanded clonotypes. Our first-in-human trial demonstrated its safety and potentially preliminary efficacy, warranting further clinical evaluation. Multiomic profiling identified specific circulating NK and T-cell subsets potentially associated with clinical outcomes, providing novel insights into the dynamic transcriptional underpinnings of the immune landscape in response to NK cell-based therapy.

## Introduction

Pancreatic cancer (PC) is one of the most lethal human malignancies, with a five-year survival rate of less than 10%. As pancreatic cancers are typically asymptomatic during the early stages, more than 90% of patients are diagnosed at an advanced stage.^1^ Currently, the treatment options for advanced pancreatic cancer (APC) are exceedingly limited, and systemic chemotherapy remains the standard therapy. In Asian countries, gemcitabine plus tegafur/gimeracil/oteracil potassium (S-1) is recommended as first-line chemotherapy owing to the increased prevalence of low-activity CYP2A6 phenotypes in Asian populations, a pharmacogenetic feature that enhances the therapeutic efficacy of S-1.^2–4^ Nonetheless, notwithstanding its application, this combination chemotherapy in APC exhibited only moderate effectiveness and elicited concerns regarding the potential risk of toxicity and drug resistance, collectively contributing to poor prognosis.^5^ Consequently, novel therapeutic strategies against pancreatic cancer are urgently needed.

Recent advances in cancer immunotherapy have revolutionized the landscape of cancer therapy,^6^ with immune checkpoint inhibitors (ICIs) demonstrating remarkable efficacy in multiple malignancies by reinvigorating T-cell-mediated antitumor responses.^7^ However, the effectiveness of ICIs in treating PC remains disappointing, as the immunosuppressive tumor microenvironment (TME), which is characterized by dense stromal barriers and dysfunctional endogenous T cells, renders ICIs largely ineffective.^8^ These treatment challenges have spurred interest in alternative strategies beyond immune checkpoint inhibitors in PC, among which natural killer (NK) cell-based therapies have emerged as promising options.^9^ Unlike T lymphocyte-based therapies, NK cells exhibit strong cytotoxic activity against tumor cells without prior sensitization or immunization, and their “off-the-shelf” feasibility further enhances their clinical applicability.^10^ Preclinical studies in PC have demonstrated that chimeric antigen receptor (CAR)-NK cells or cytokine-activated NK-92 cell lines effectively lyse tumor cells,^11,12^ while synergizing with gemcitabine efficiently inhibits pancreatic tumors both in vitro and in vivo.^13,14^ Early-phase trials involving adoptive NK cell transfer have also indicated a favorable safety profile and preliminary efficacy in hematological malignancies and hepatocellular carcinoma.^15,16^

Despite these advancements, the clinical translation of NK cell therapy in PC remains in the preclinical stage or ongoing early-phase trials, with only fragmented clinical outcomes reported.^17^ Although combinatorial strategies incorporating chemotherapy have shown promise in other malignancies,^15,18,19^ no clinical evidence exists for combining NK cell therapy with gemcitabine plus S-1 (GS) for the treatment of pancreatic cancer. Moreover, investigations into NK cell therapy have focused primarily on individual biomarkers, such as post-infusion increases in circulating NK cell counts or cytotoxic cytokine levels.^17,20^ However, no study has comprehensively investigated the dynamic peripheral immune milieu in response to NK cell therapy in PC, especially with respect to single-cell insight.

In this study, we initiated a first-in-human, single-arm, nonrandomized, phase 1b/2 clinical trial (ChiCTR1900021764) in patients with advanced pancreatic cancer receiving GS chemotherapy to evaluate the safety and feasibility of allogeneic NK cell therapy. Additionally, during the course of the trial, we collected 19 peripheral blood samples from seven patients for single-cell RNA sequencing (scRNA-seq) and bulk T-cell receptor (TCR) Vβ repertoire sequencing, aiming to characterize the dynamic immune landscapes comprehensively in patients with distinct clinical outcomes, offering novel insights into immune microenvironment reconstitution following NK cell combination therapy.

## Results

### Clinical trial design and patient characteristics

**Fig. 1:**
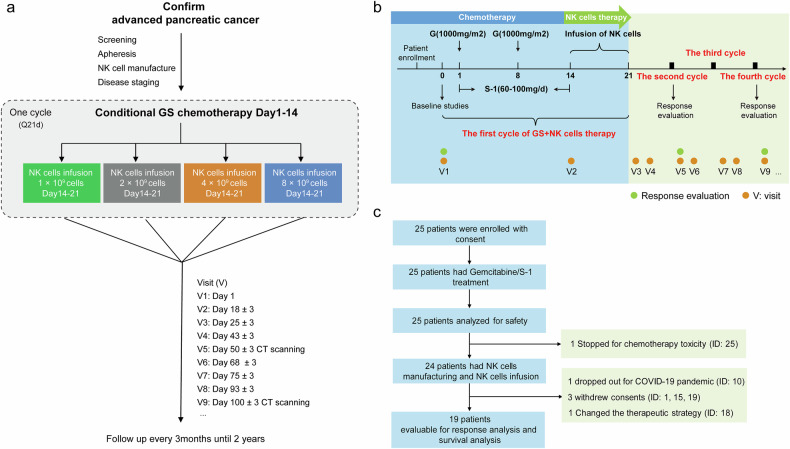
Study procedure schema and Consolidated Standards of Reporting Trials (CONSORT) diagram. **a** Clinical trial schema for screening, apheresis, NK cell manufacturing, treatment with chemotherapy combined with NK cell infusion, and follow-up. **b** Flowchart of the detailed clinical treatment protocol. Each treatment cycle included standard GS chemotherapy from days 1 to 14, followed by allogeneic NK cell administration from days 14 to 21 (a single infusion per cycle for doses of 1 × 10^9^ cells, 2 × 10^9^ cells, and 4 × 10^9^ cells, or half of the dose fractionated over two consecutive days for a dose of 8 × 10^9^ cells). **c** CONSORT flow diagram indicating the number of patients enrolled, who received treatments, and who were included in the analysis. G gemcitabine, GS gemcitabine plus S-1, V visit, CT computed tomography

Adult APC patients scheduled to receive GS as first-line systemic therapy were recruited. Patients were arranged to receive activated allogeneic NK cells in a dose-escalation design (Fig. 1a). Each treatment cycle consisted of standard GS chemotherapy on days 1 to 14, followed by a single allogeneic NK cell infusion between days 14 and 21. Patients were scheduled to receive 6 to 8 cycles of GS chemotherapy followed by S-1 maintenance, with NK cells administered during the first four cycles. Patients who expressed a strong desire to continue NK cell therapy beyond four cycles were eligible for compassionate treatment, with the treatment cycle frequency determined by the investigators on the basis of individual tolerance and response (Fig. 1b).

From March 8, 2019, to September 16, 2022, 25 patients signed informed consent forms and were followed up until July 30, 2023. Among these 25 patients, 24 received NK cell infusion treatment (1 patient dropped out due to severe chemotherapy toxicity), and 19 underwent response analysis and survival assessment (1 patient dropped out due to the COVID-19 pandemic, and 4 patients withdrew consent or changed the therapeutic strategy before remission was confirmed) (Fig. 1c). The time of study for each patient and the duration of follow-up off the clinical trial (survival since the time of enrollment, n = 24) are indicated in Supplementary Fig. 1.

The clinical characteristics of the 25 enrolled APC patients are listed in Table 1 and Supplementary Table 1. The median age was 64 (44–75) years, and 80.0% (20/25) of the patients were men. Three patients (12.0%) had locally advanced pancreatic cancer (LAPC), 22 patients (88.0%) had metastatic pancreatic cancer (MPC), and 32.0% (8/25) of them had at least three metastatic sites involved. Twenty-four (96.0%) patients received at least one cycle of NK cell-based therapy, 9 of whom were infused with peripheral blood (PB)-derived NK cells and 15 of whom were infused with cord blood (CB)-derived NK cells. All the allogeneic NK cell products met the predefined release criteria, and no significant differences in purity, viability or cytotoxicity were observed between the PB- and CB-derived NK cell products (Supplementary Fig. 2). The NK cell dose levels (DLs) of 1 × 10^9^, 2 × 10^9^, 4 × 10^9^ and 8 × 10^9^ NK cells were administered to 3, 5, 6 and 5 patients, respectively. All these DL cohorts did not significantly differ with respect to demographic characteristics.

**Table 1:** Clinical characteristics of all patients (*n* = 25)

Characteristic	1 × 10^9^ (*n* = 4)	2 × 10^9^ (*n* = 6)	4 × 10^9^ (*n* = 7)	8 × 10^9^ (*n* = 7)	NA^a^ (*n* = 1)	All (*n* = 25)
Median age (range), year	61 (44–64)	67 (56–75)	58 (51–65)	68 (48–71)	69	64 (44–75)
Gender, *n* (%)						
Female	2 (50.0)	2 (33.3)	0	1 (14.3)	0	5 (20.0)
Male	2 (50.0)	4 (66.7)	7 (100)	6 (85.7)	1 (100)	20 (80.0)
Disease status, *n* (%)						
Locally advanced	0	1 (16.7)	1 (14.3)	1 (14.3)	0	3 (12.0)
Metastatic	4 (100)	5 (83.3)	6 (85.7)	6 (85.7)	1 (100)	22 (88.0)
Site of primary tumor, *n* (%)						
Body and Tail	2 (50.0)	3 (50.0)^b^	6 (85.7)	5 (71.4)	0	16 (64.0)^b^
Head and neck	2 (50.0)	3 (50.0)	1 (14.3)	2 (28.6)	1 (100)	9 (36.0)
Number of metastatic organs, *n* (%)						
≤2	3 (75.0)	6 (100)	2 (28.6)	5 (71.4)	1 (100)	17 (68.0)
≥3	1 (25.0)	0	5 (71.4)	2 (28.6)	0	8 (32.0)
Liver metastasis						
No	2 (50.0)	3 (50.0)	4 (57.1)	2 (28.6)	0	11 (44.0)
Yes	2 (50.0)	3 (50.0)	3 (42.9)	5 (71.4)	1 (100)	14 (56.0)
Peritoneal metastasis						
No	3 (75.0)	5 (83.3)	3 (42.9)	6 (85.7)	1 (100)	18 (72.0)
Yes	1 (25.0)	1 (16.7)	4 (57.1)	1 (14.3)	0	7 (28.0)
Lung metastasis						
No	3 (75.0)	6 (100)	6 (85.7)	6 (85.7)	1 (100)	22 (88.0)
Yes	1 (25.0)	0	1 (14.3)	1 (14.3)	0	3 (12.0)
Lymph node metastasis						
No	3 (75.0)	4 (66.7)	2 (28.6)	4 (57.1)	0	13 (52.0)
Yes	1 (25.0)	2 (33.3)	5 (71.4)	3 (42.9)	1 (100)	12 (48.0)
NK cells Infusion Cycles, *n* (%)						
0	0	0	0	0	1 (100)	1 (4.0)
1	0	1 (16.7)	1 (14.3)	0	0	2 (8.0)
2	0	0	2 (28.6)	3 (42.9)	0	5 (20.0)
3	1 (25.0)	1 (16.7)	1 (14.3)	1 (14.3)	0	4 (16.0)
≥4	3 (75.0)	4 (66.7)	3 (42.9)	3 (42.9)	0	13 (52.0)

^a^NA, not available

^b^Tumoral sites of patient P12 located both in neck, body and tail

### Safety and efficacy in the dose‒response relationship

The primary endpoint was the safety and tolerability of the NK cell combination therapy. A total of 56.0% of patients reported Grade 3 or higher treatment-related adverse events (TRAEs), including neutropenia, leukopenia, thrombocytopenia, anemia, rash, and increased alanine aminotransferase, alkaline phosphatase, and γ-glutamyl transferase, which were mainly related to chemotherapy (Supplementary Table 2, Supplementary Fig. 3a). A total of 8.0% of patients (2/25, P15 and P27) experienced chemotherapy-related severe adverse events, presenting as grade 4 neutropenia. Notably, none of the patients necessitated admission to the intensive care unit for the management of adverse events associated with combination therapy (Supplementary Table 3).

No predefined dose-limiting toxicities (DLTs) with grade 3 or higher TRAEs were observed during NK cell infusion. All the NK-cell infusion-related TRAEs were Grade 2 or lower, including fever (4/24, 16.7%), fatigue (3/24, 12.5%), chill (2/24, 8.3%), and rash (1/24, 4.2%), and no graft-versus-host disease (GVHD) was observed (Table 2, Supplementary Fig. 3b). In addition, 20.0% of the NK cell infusion-related TRAEs occurred in cohort 1 (fever, 2/10, P3 and P4), 10.0% occurred in cohort 2 (fatigue, 1/10, P9), and 70.0% of the events occurred in cohort 4 (fever, fatigue, chill and rash, 7/10, P22, P26 and P27) (Supplementary Fig. 3c). All Grade 1 TRAEs appeared several hours after the infusion and resolved spontaneously overnight without intervention. Specifically, only one patient (P22), whose highest body temperature reached 39.5 °C accompanied by moderate whole-body tremor, experienced Grade 2 fever and chills within 2 hours after each infusion. The patient recovered after 3 hours following antipyretic and antiallergic treatment.

**Table 2:** Treatment-related adverse events reported with infusion of NK cells (*n* = 24^a^)

Preferred term^b^, *n* (%)	Cohort 1		Cohort 2		Cohort 3		Cohort 4		All cohort	
	(1 × 10^9^, *n* = 4)		(2 × 10^9^, *n* = 6)		(4 × 10^9^, *n* = 7)		(8 × 10^9^, *n* = 7)		(*n* = 24)	
	Grade1–2	Grade3-4	Grade1–2	Grade3-4	Grade1–2	Grade3-4	Grade1–2	Grade3-4	Grade1–2	Grade3-4
Fever	2 (50.0)	0	0	0	0	0	2 (28.6)	0	4 (16.7)	0
Fatigue	0	0	1 (16.7)	0	0	0	2 (28.6)	0	3 (12.5)	0
Chill	0	0	0	0	0	0	2 (28.6)	0	2 (8.3)	0
Rash	0	0	0	0	0	0	1 (14.3)	0	1 (4.2)	0
GVHD^c^	0	0	0	0	0	0	0	0	0	0

^a^P25 withdrew from the clinical trial without receipting NK cells treatment due to the chemotherapy toxicity

^b^Medical Dictionary for Regulatory Activities version 23.1, graded according to CTCAE version 5.0

^c^GVHD, Graft-versus-host disease

**Fig. 2:**
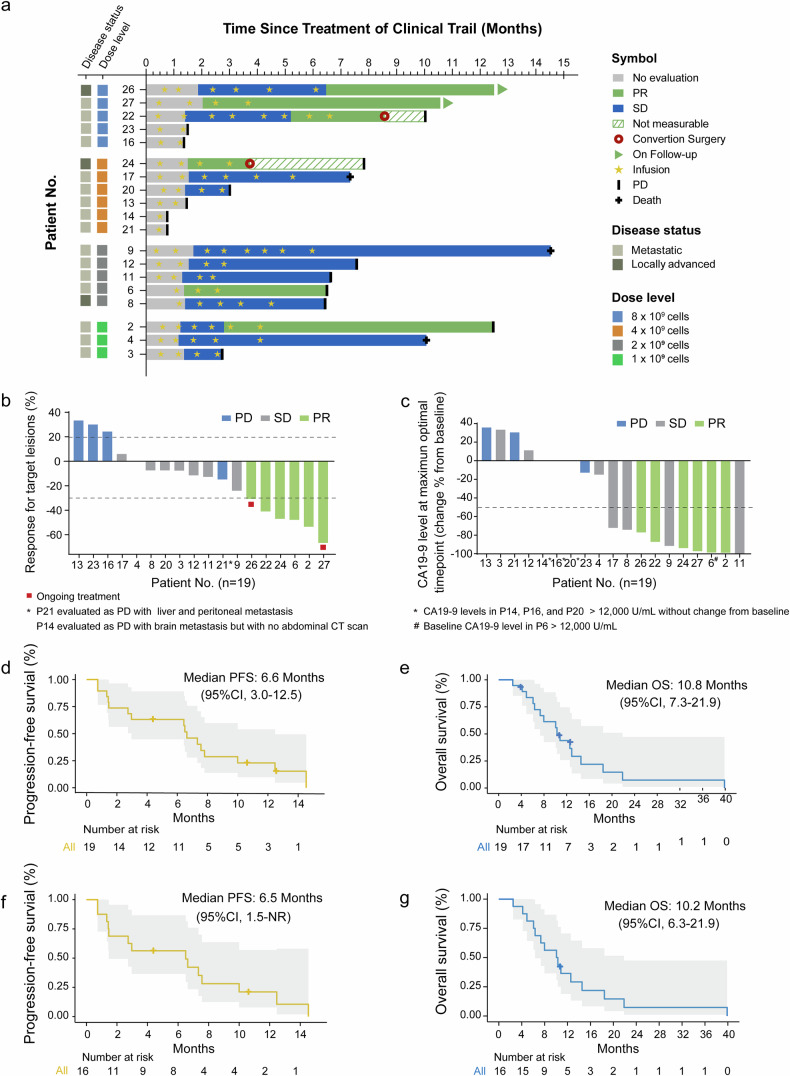
Duration of response, objective response, and patient survival after NK cell-based combination therapy. **a** Swimmer plot for each patient, describing the characteristics and clinical outcomes, grouped by dose. The best response was confirmed and assessed according to RECIST 1.1 by investigators (*n* = 19). **b** Waterfall plot showing the maximum change in the target lesion from baseline (*n* = 18). P14 was excluded because of failure to assess the target lesions and was assessed as having progressive disease (PD) with new brain metastasis prior to the first efficacy assessment. P21 included target lesion shrinkage despite being diagnosed with PD due to new liver and peritoneal metastases. **c** Waterfall graph depicting the maximum percentage change in the serum CA19-9 level (50% decrease in the CA19-9 level marked with a dashed line) after the combination treatment. *, patients whose CA19-9 level was greater than the threshold are marked (P6, P14, P16 and P20). Kaplan‒Meier curves for (**d**) PFS and (**e**) OS among advanced pancreatic cancer (APC) patients since enrollment (*n* = 19). The risk table shows the number of patients with ongoing survival at each time point. The median follow-up was 17.7 months (range 2.5–39.9) according to the reverse Kaplan–Meier method. Kaplan‒Meier curves for (**f**) PFS and (**g**) OS among patients with metastatic pancreatic cancer (MPC) since enrollment (*n* = 16). Risk table showing the number of patients with ongoing survival at each time point. PR partial response, SD stable disease, PD progressive disease, PFS progression-free survival, OS overall survival, CT computed tomography

For preliminary efficacy, 19 APC patients were evaluated for clinical response, with a median follow-up period of 17.7 months (range 2.5–39.9). The median number of NK cell infusion cycles for each individual was 4, ranging from 1–13. Across all four dose-escalation cohorts, 6 patients (31.6%) achieved partial response (PR), and 8 patients (42.1%) achieved stable disease (SD) (Fig. 2a, Table 3 and Supplementary Table 4). Eighteen subjects had measurable postbaseline target lesions; among them, 13 patients (72.2%) experienced tumor regression (Fig. 2b). The objective response rate (ORR) and disease control rate (DCR) were 31.6% (95% CI, 12.6–56.6) and 73.7% (95% CI, 48.8–90.9), respectively (Table 3). When measured by the maximum decline in CA19-9 levels from baseline, 10 out of 19 treated subjects (52.6%) demonstrated a reduction of at least 50% in CA19-9 levels, which was consistent with the observed decrease in CA19-9 levels among patients with PR and SD (Fig. 2c). The median progression-free survival (mPFS) and median overall survival (mOS) reached 6.6 months (95% CI, 3.0–12.5) and 10.8 months (95% CI, 7.3–21.9), respectively (Fig. 2d, e and Table 3).

**Table 3:** Efficacy outcomes of advanced pancreatic cancer patients by investigator assessment

Responses (RECIST v1.1)	Advanced pancreatic cancer (*n* = 19)	Metastatic pancreatic cancer (*n* = 16)
Best overall response,		
PR, No. (%)	6 (31.6)	4 (25.0)
SD, No. (%)	8 (42.1)	7(43.8)
PD, No. (%)	5 (26.3)	5 (31.2)
ORR, No. (%) [95% CI]	6 (31.6) [12.6, 56.6]	4 (25.0) [7.3, 52.4]
DCR, No. (%) [95% CI]	14 (73.7) [48.8, 90.9]	11 (68.8) [41.3, 89.0]
mPFS(months) [95% CI]	6.6 [3.0, 12.5]	6.5 [1.5, NR]
6-months PFS rate (%) [95% CI]	57.4 [38.8, 84.9]	49.2 [29.7, 81.6]
mOS(months) [95% CI]	10.8 [7.3, 21.9]	10.2 [6.3, 21.9]
6-months OS rate (%) [95% CI]	78.0 [61.1, 69.3]	75.0 [56.5, 99.5]

*CR* complete response, *DCR* disease control rate, *NA* not applicable, *ORR* objective response rate, *PD* progressive disease, *PR* partial response, *SD* stable disease

For MPC patients (*n* = 16), the ORR and DCR reached 25.0% (95% CI, 7.3–52.4) and 68.8% (95% CI, 41.3–89.0), respectively. For long-term efficacy, the mPFS of MPC patients was 6.5 (95% CI, 1.5-not reached, NR) months, and the mOS was 10.2 (95% CI, 6.3–21.9) months (Fig. 2f, g and Table 3). In addition, two patients who achieved a PR (P22 and P24) underwent conversion surgery (surgical resection after combination therapy) after achieving a satisfactory response, with recurrence-free survival times of 1.5 months and 4.1 months, respectively (Fig. 2a).

### Single-cell transcriptional profiles: distinct immune cell signatures associated with clinical response

**Fig. 3:**
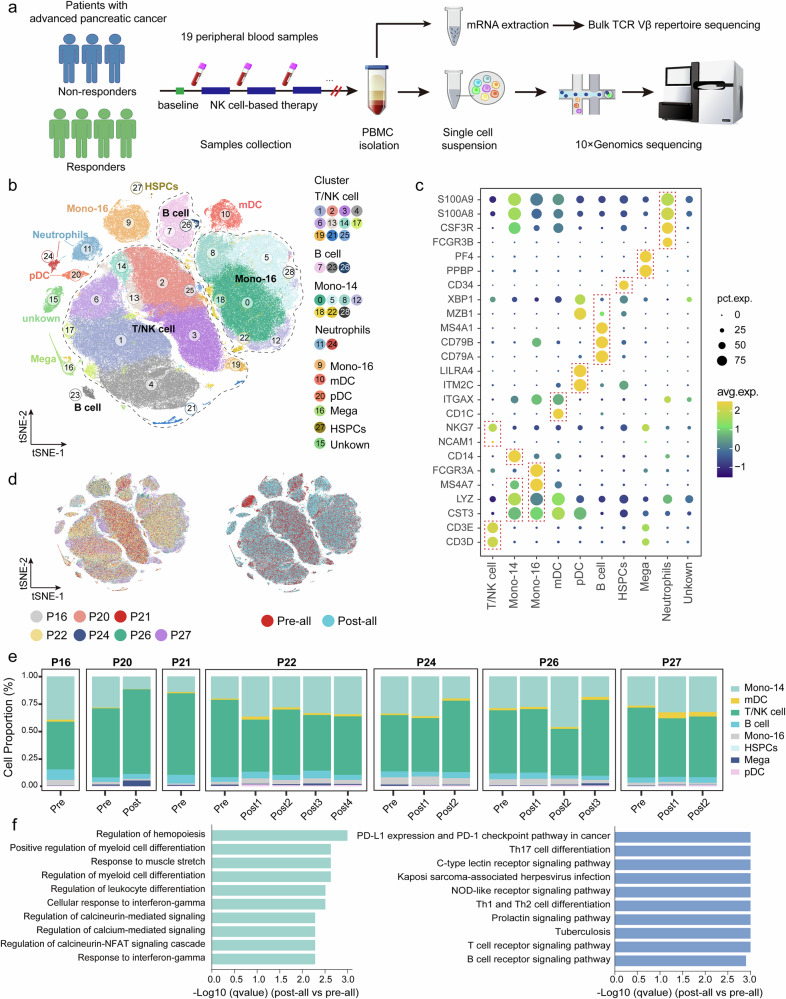
Single-cell transcriptional profiling of immune cells in APC patients treated with NK cell-based therapy. **a** Schematic overview of the study design. Three nonresponders (NR, including the SD/PD samples P16, P20, and P21) and four responders (R, including the CR/PR samples P22, P24, P26, and P27) were sampled for scRNA-seq. Three responders (P24, P26, and P27) were enrolled for bulk TCR Vβ repertoire sequencing. **b** t-SNE plot visualization of circulating immune cell clusters. **c** Dot plot showing the expression levels of marker genes in the indicated immune cell types. **d** t-SNE plot showing the cell origins by color. Patient origin is shown in the left panel, and pretreatment (pre–all, *n* = 7) or posttreatment (post–all, *n* = 12) data are shown in the right panel. **e** Histogram indicating the proportion of annotated immune cells in each sample; neutrophils and unknown cells were excluded from the proportion analysis. **f** GO and KEGG analyses of differentially expressed genes (DEGs) between pre–all and post–all samples. DEGs were computed by *Findmarkers* with a |log2-fold change| > 0.25 and adjusted *p* value < 0.05. The top 10 enriched GO-BP terms (left panel) and the top 10 enriched KEGG pathways (right panel) upregulated in all the samples are shown. scRNA-seq single-cell RNA sequencing, TCR T-cell receptor, t-SNE t-distributed stochastic neighbor embedding, GO gene ontology, KEGG Kyoto Encyclopedia of Genes and Genomes, GO-BP GO-biological process

The exploration endpoint was to estimate the underlying dynamic peripheral immune landscape in response to NK cell-based therapy in APCs. Seven APC patients (including 4 responders and 3 nonresponders) were enrolled for scRNA-seq, and three of the responders underwent bulk TCR Vβ repertoire sequencing. Nineteen longitudinal peripheral blood mononuclear cell (PBMC) samples were collected from multiple timepoints across clinical trial cycles, followed by droplet-based scRNA-seq (10× Genomics platform) (Fig. 3a and Supplementary Fig. 4a). A total of 190,041 immune cells were subjected to further analyses. Twenty-eight clusters were identified and visualized via the t-distributed stochastic neighbor embedding (t-SNE) method (Fig. 3b), and these cell populations were then divided into T/NK cells, B cells, neutrophils, CD14^+^ monocytes, CD16^+^ monocytes, monocyte-derived dendritic cells (mDCs), plasmacytoid dendritic cells (pDCs), megakaryocytes, and hematopoietic stem and progenitor cells (HSPCs) on the basis of their canonical cell marker genes^21,22^ (Fig. 3c and Supplementary Fig. 4b, c). All of these cell subtypes were shared among patients and between pre- and posttreatment samples, indicating a well-corrected batch effect (Fig. 3d). Notably, neutrophils were excluded from downstream analyses because of technical deviations. The results revealed that the populations of circulating T/NK cells and CD14^+^ monocytes were relatively high in all patients with APC (Fig. 3e).

We then analyzed differentially expressed genes (DEGs) between all pre- and posttreatment samples. Gene Ontology (GO) and Kyoto Encyclopedia of Genes and Genomes (KEGG) analyses revealed that the genes highly expressed in patients after treatment were involved mainly in pathways related to immune cell differentiation, the response to interferon-gamma, T-cell receptor signaling and B-cell receptor signaling (Fig. 3f). In addition, gene set enrichment analysis (GSEA) revealed significant enrichment of hallmark pathways associated with the inflammatory response and proinflammatory cytokines in posttreatment patient samples (Supplementary Fig. 4d). These results suggest that APC patients exhibit an enhanced overall peripheral immune response after receiving NK cell-based therapy.

### Efficacy-associated circulating NK cell subtypes expanded in response to NK cell-based therapy

**Fig. 4:**
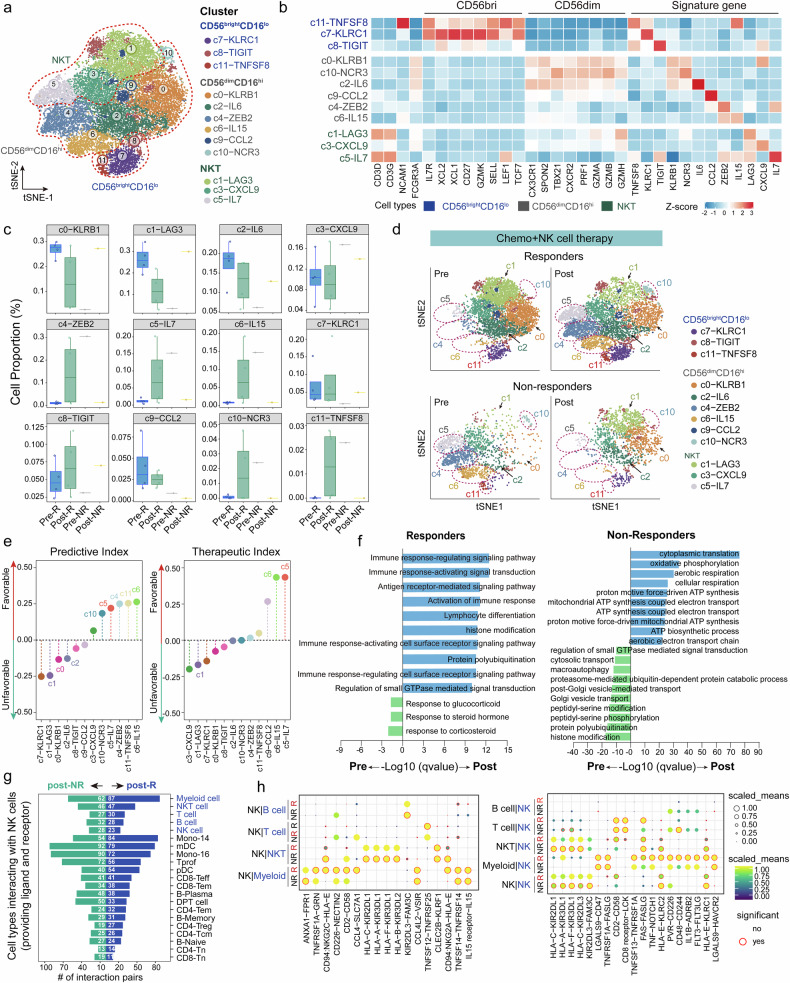
Characteristics and temporal dynamics of circulating NK/NKT cell subtypes following NK cell-based therapy. **a** t-SNE plot visualization of CD56^bright^CD16^lo^ NK cells, CD56^dim^CD16^hi^ NK cells, and NKT cells derived from APC patients. **b** Heatmap showing the expression patterns of functional genes and signature genes of each cluster. The color is coded by the Z score-scaled gene expression. Boxplots (**c**) and t-SNE plots (**d**) showing the temporal alterations in the NK/NKT cell clusters. pre-R, baseline samples of responders (*n* = 4, P22-pre, P24-pre, P26-pre, and P27-pre); post-R, posttreated samples of responders at the timepoint of the first observation of PR (*n* = 4, P22-post3, P24-post1, P26-post3, and P27-post1); pre-NR, baseline samples of nonresponder (*n* = 1, P20-pre); post-NR, posttreated sample of the nonresponder when PD was reached (*n* = 1, P20-post). Box middle lines, median; box limits, upper and lower quartiles; box whiskers, 1.5× the interquartile range. **e** Pi and Ti analysis of NK/NKT cell clusters. Pi, predictive index; Ti, therapeutic index. The Pi quantifies the correlation between baseline immune cell proportions and initial tumor size, whereas the Ti assesses the correlations between posttreatment immune cell proportions and changes in tumor size. **f** GO analysis of DEGs between pre- and posttreatment samples from responders (*n* = 4, left panel) and nonresponders (*n* = 1, right panel). DEGs were computed by *Findmarkers* with a |log2-fold change| > 0.5 and adjusted *p* value < 0.05. The top 10 enriched GO-BP terms enriched in pre- (green column) or posttreatment (blue column) samples are shown. **g** Bar chart showing the number of potentially significant ligand‒receptor pairs in NK cells (including CD56^bright^CD16^lo^ NK cells and CD56^dim^CD16^hi^ NK cells) and other immune cells in post-NR (*n* = 1) and post-R (*n* = 4) samples. The number of NK cells that provided ligands and receptors were calculated together. **h** Bubble chart showing the significantly upregulated interaction pairs in post-NR (*n* = 1) or post-R (*n* = 4) samples. The color and size of bubbles represent the mean expression of ligand and receptor genes, and the bubbles with a red circle indicate the statistical significance of interactive molecular pairs, as calculated by CellPhoneDB

We conducted two rounds of unsupervised clustering for the NK cells. The first round of analysis aimed to distinguish T cells from NK cells on the basis of the expression levels of the canonical cell markers *NCAM1* and *FCGR3A*. In the second round of unsupervised clustering, 20,620 NK cells were further subdivided into 12 subsets (c0-c11), including CD56^bright^CD16^lo^ NK cells, CD56^dim^CD16^hi^ NK cells, and NKT cells,^23–25^ on the basis of the high expression of a unique signature gene within each cluster (Fig. 4a and Supplementary Fig. 5a, b). To better understand the characteristics of peripheral immune cells in response to NK cell-based therapy, we purposely included paired cases and enrolled their samples at the time of the first observation of PR for subsequent dynamic analyses. We found that the proportion of CD56^dim^CD16^hi^ NK cells decreased after treatment in nonresponders, whereas that in responders tended to increase (Supplementary Fig. 5c). To systematically evaluate the associations between distinct immune cell subtypes and clinical responses, we employed two analytical indices: the predictive index (Pi) and the therapeutic index (Ti), modified from previous studies.^26^ A positive Pi or Ti indicates that a higher baseline or posttreatment proportion of the corresponding immune cell subtype is associated with a smaller baseline tumor size or greater tumor shrinkage following treatment, thus predicting or mediating a favorable clinical response, respectively. Here, Pi and Ti analyses also suggested a potential association between CD56^dim^CD16^hi^ NK cells and favorable clinical outcomes (Supplementary Fig. 5d). Consistently, a higher level of CD56^dim^CD16^hi^ NK cells after treatment appeared to be correlated with longer patient survival (Supplementary Fig. 5e).

Previously reported scRNA-seq signatures of NK cell subtypes,^25,27^ including active, mature, adaptive, and human leukocyte antigen^high^ (HLA^hi^) NK cells, were also partially identified in our data. Among these peripheral atlases, several underappreciated expression signatures of these NK-cell subtypes have been discovered. Our data revealed that the CD56^bright^CD16^lo^ NK cell subsets and certain CD56^dim^CD16^hi^ NK cell subsets (c6-IL15, c9-CCL2, and c10-NCR3) exhibited active NK-like functions (*CD69*, *DUSP1*, *FOS*, and *JUN)*. The NKT cell subsets, as well as the c4-ZEB2, c10-NCR3, and c8-TIGIT cell clusters, potentially function like adaptive NK cells (*KLRC2*, *CD52*, and *IL32*) (Supplementary Fig. 5f). Next, we evaluated the potential correlation between NK cell cluster dynamics and clinical outcomes. Compared with nonresponders, responders exhibited consistent increases in the proportions of clusters c4-ZEB2, c5-IL7, c6-IL15, c10-NCR3, and c11-TNFSF8 following NK cell-based therapy, accompanied by a reduction in clusters c0-KLRB1, c1-LAG3, and c2-IL6 (Fig. 4c, d). Consistently, the Pi analysis suggested that clusters c4-ZEB2, c5-IL7, c6-IL15, c10-NCR3, and c11-TNFSF8, rather than c0-KLRB1, c1-LAG3 and c2-IL6, were correlated with favorable outcomes. The Ti analysis partially supported that the expansion of c5-IL7 and c6-IL15, but not c1-LAG3, was associated with a favorable response following treatment. To elucidate the functional heterogeneity underlying these clinical associations, we examined the gene expression profiles across clusters. Notably, clusters c0-KLRB1, c1-LAG3, or c2-IL6 presented increased expression of *KLRC1*, *KLRB1*, *KLRG1*, *LAG3*, and *HAVCR2*, suggesting an inhibitory and exhausted phenotype. In contrast, although c4-ZEB2, c5-IL7, c6-IL15, c10-NCR3, and c11-TNFSF8 sporadically expressed the inhibitory receptors KLRB1 and KLRC1, respectively, they were characterized by increased expression of proinflammatory and effector molecules, including cytokines (*IL15*, *IL7*, and *IFNG*), coreceptors (*CD244*, *CD226*, *SLAMF6*, and *TNFSF8*), and activation-related genes (*KLF12*, *NCR1*, *IL7R*, and *IL12RB2*) (Supplementary Fig. 5g). To further validate these findings, we analyzed an independent published scRNA-seq dataset from breast cancer patients treated with a PD-L1 inhibitor plus chemotherapy (GSE266919).^28^ Notably, despite differences in therapeutic regimens (NK cell therapy *vs*. PD-L1 inhibitor) and tumor types (pancreatic cancer *vs*. breast cancer), we still observed that c4-ZEB2 and c10-NCR3 exhibited greater expansion patterns in responders than in nonresponders (Supplementary Fig. 6a, b), which was consistent with the trends in our dataset.

### NK cells activated immune transcriptional programs in responsive patients

By comparing the transcriptomic differences in circulating NK cells between responders and nonresponders, we found that posttreated NK cells in responders were highly enriched in GO terms related to immune response regulation, activation, and lymphocyte differentiation. In contrast, nonresponders were sporadically involved in the immune response but were instead involved in cytoplasmic translation, oxidative phosphorylation, or aerobic respiration (Fig. 4f).

Next, we sought to evaluate the differences in communication between NK cells and immune cells before and after treatment. The results revealed that NK cells exhibited closer interactions with myeloid cells in posttreated responders (Fig. 4g and Supplementary Fig. 5h). When we focused on the predicted ligand‒receptor pairs, intensive cytotoxicity chemokines, costimulatory interactions, and activation-related interactions, such as TNFSF13‒TNFRSF1A, FAS‒FASLG, PVR‒CD226, TNFRSF1A‒GRN, and HLA‒E‒KLRC2, were significantly different between NK cells and myeloid cells in posttreatment responders. Nonresponders were significantly enriched in killer cell immunoglobulin-like receptor (KIR) inhibitory receptor interactions between NK self-interactions or interactions with NKT cells (Fig. 4h). Collectively, these observations suggest that NK cell subsets may be positively associated with favorable clinical outcomes in response to NK cell-based immunotherapy, potentially through an activated immune transcriptional program.

### Circulating T cells display an enhanced immune response and clonal expansion in response to combination therapy

**Fig. 5:**
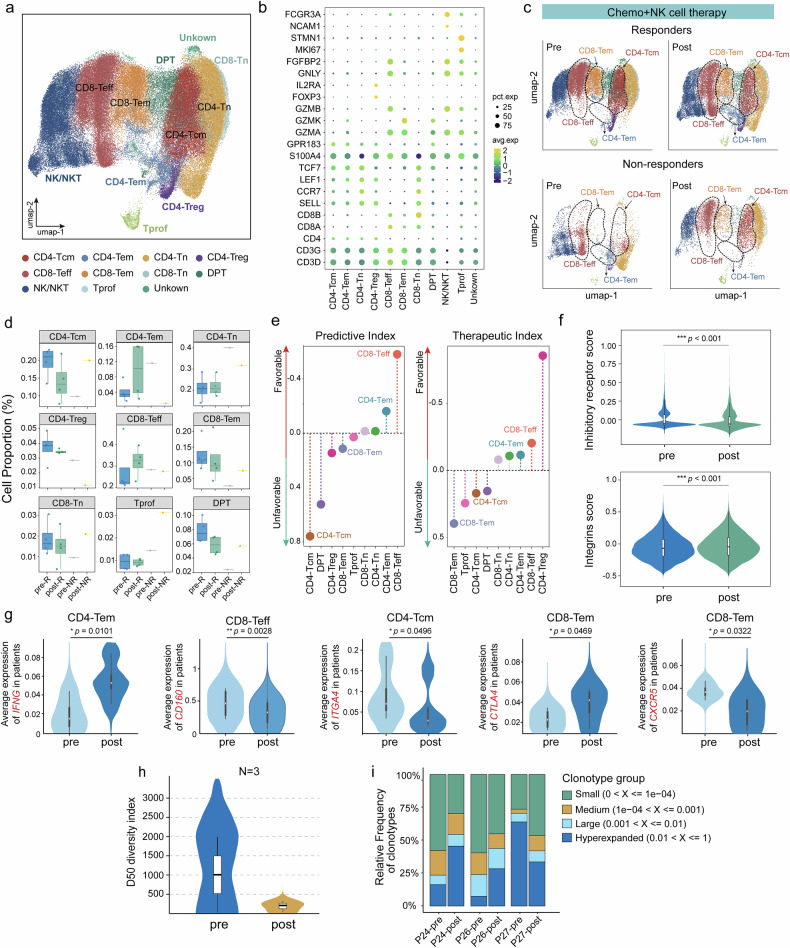
Characteristics and temporal dynamics of T-cell subsets in response to NK cell-based therapy. **a** UMAP of subclustered T/NK cells, labeled in distinct colors. Cell type annotations are shown. **b** Dot plot showing the expression levels of marker genes in the indicated T-cell subsets or NK/NKT cells. UMAP plots (**c**) and boxplots (**d**) showing the distribution and dynamic alteration of T-cell subtypes following NK cell-based therapy. Box middle lines, median; box limits, upper and lower quartiles; box whiskers, 1.5 × the interquartile range. **e** Pi and Ti analysis of T-cell subsets. **f** Violin plots showing the dynamic alterations in the inhibitory score and integrin score of T cells in responders (*n* = 4). The *p* value was calculated via the Wilcoxon test. **g** Violin plots indicating the dynamic alterations in the average expression of significant genes in specific T-cell subsets in responders (*n* = 4). The *p* value was calculated via a two-tailed paired Student’s t test. **h** Comparison of the D50 diversity indices of the TCR Vβ repertoire in the peripheral blood of pre- (*n* = 3, P24-pre, P26-pre, and P27-pre) or posttreated responders (*n* = 3, P24-post1, P26-post3, and P27-post1). A low D50 diversity index indicates high clonal expression and low diversity. **i** Distribution of four clonotype groups in pre- or posttreated responders (defined on the basis of the relative frequency of clonotypes). A *p* value < 0.05 was considered statistically significant (*p < 0.05, **p < 0.01, ***p < 0.001). UMAP, uniform manifold approximation and projection

To gain further insight into the dynamic response of T cells following treatment, we subclustered T/NK cells and identified nine canonical T-cell groups: 4 subsets of CD4^+^ T cells, including central memory (CD4^+^ Tcm), effector memory (CD4^+^ Tem), naïve (CD4^+^ Tn) and regulatory (Treg) T cells; 3 subsets of CD8^+^ T cells, including effector (CD8^+^ Teff), effector memory (CD8^+^ Tem) and naïve (CD8^+^ Tn) T cells; one subtype of double-positive T cells, one subtype of proliferating T cells (Fig. 5a, b). In responsive patients, we observed increased proportions of CD4^+^ Tems and CD8^+^ Teffs after combination treatment, in contrast with the increased proportions of CD4^+^ Tcms and CD8^+^ Tems in nonresponsive patients. Pi and Ti analyses partly supported this dynamic signature, indicating that the expansion of CD4^+^ Tem and CD8^+^ Teff cells was correlated with favorable responses, whereas increased proportions of CD4^+^ Tcm and CD8^+^ Tem cells were associated with unfavorable clinical outcomes (Fig. 5e). Validation via a breast cancer scRNA-seq dataset (GSE266919)^28^ from patients treated with a PD-L1 inhibitor plus chemotherapy revealed concordant patterns. Importantly, both the CD4^+^ Tem and CD8^+^ Teff populations expanded more in responders than in nonresponders did (Supplementary Fig. 6c-f), which was consistent with the T-cell dynamics observed in our dataset.

A comparison of the transcriptional dynamics of treatment-induced T cells via GSEA revealed that T cells from treatment responders were significantly enriched in pathways related to cytotoxic cytokine production, inflammation, and T-cell activation and differentiation (Supplementary Fig. 7a, b). Additionally, T cells exhibited a significantly decreased inhibitory receptor score and increased integrin score in responsive patients (Fig. 5f), whereas the opposite pattern was observed for nonresponding T cells (Supplementary Fig. 7c). We also observed that the potential response-related T-cell subsets were characterized by upregulation of the effector function-related gene *IFNG* in CD4^+^ Tems, downregulation of the inhibitory receptor gene *CD160* in CD8^+^ Teffs, and decreased expression of the integrin gene *ITGA4* and the chemokine receptor *CXCR5* in CD4^+^ Tcms and CD8^+^ Tems, respectively. Additionally, CD8^+^ Tems presented elevated expression of the inhibitory receptor *CTLA4* (Fig. 5g).

Bulk TCR Vβ repertoire sequencing was further performed to evaluate the clonal expansion of T cells in response to treatment. In three responders (P24, P26, and P27), we observed a decrease in T-cell diversity (measured as the D50 diversity index) after treatment (Fig. 5h), suggesting potential clonal expansion of circulating T cells upon NK cell-based therapy. This finding was supported by an increased enrichment of large and hyperexpanded clonotypes (with a relative abundance > 0.001) (Fig. 5i), as well as a greater contribution of the top 100 clonotypes to the entire repertoire after treatment (Supplementary Fig. 7d). Notably, patient P27, who responded well to the therapy, presented a greater baseline proportion of expanded TCR clonotypes, which were maintained or further elevated throughout the therapy, suggesting that a preexisting and therapy-responsive T-cell repertoire may be linked to clinical benefit.

## Discussion

Chemotherapy has been established as the standard front-line treatment for APC, but the long-term prognosis of patients remains unsatisfactory. In this study, we conducted a phase 1b/2, first-in-human clinical trial to explore the safety and efficacy of dose-escalated allogeneic NK cell therapy in combination with GS chemotherapy in APC patients. Our treatment cohort demonstrated a favorable safety profile and preliminary clinical efficacy, suggesting a potential strategy for APC patients. Moreover, our study comprehensively characterized the single-cell transcriptomic profiles of peripheral immune cells on the basis of their distinct responses following NK cell-based combination therapy, indicating a response-related reconstitution of the peripheral immune landscape, particularly through enhanced functionality of NK cells and T cells. These findings help us to better understand the dynamic immune landscape for NK cell-based therapy in solid tumors and distinguish responders from nonresponders, establishing the stage for the development of precise, individualized NK cell-based immunotherapies.

Our results revealed that the administration of NK cells was well tolerated in patients across all DLs and that the maximum tolerated dose (MTD) was not reached. No DLTs or treatment-related severe adverse events (AEs) leading to withdrawal were reported. High-grade hematologic and gastrointestinal toxicities are predominantly related to conditional GS chemotherapy. All NK cell-related AEs were within Grade 2; among these, fever, fatigue, and chills occurred most frequently within several hours after infusion. In addition, despite the HLA disparity of allogeneic NK cell products and recipients, no GVHD was detected, which is consistent with previous reports on NK cell treatments for several hematological and solid malignancies.^10,15,29^

A pooled analysis of three randomized trials (GEST, JACCRO PC-01, and GEMSAP) evaluating first-line GS chemotherapy in Asian patients with advanced pancreatic cancer reported an mPFS of 5.78 months, an OS of 10.48 months, an ORR between 18.9% and 29.3%, and a DCR ranging from 64.2% to 79.2%.^2,30–32^ In this early-phase trial, we observed preliminary evidence of the efficacy of GS chemotherapy combined with NK cell therapy. Notably, the greater proportion of patients with metastatic pancreatic cancer in our cohort (16/19, 84%) may have been correlated with an underestimation of the treatment efficacy. Within this metastatic subgroup, we observed numerical improvements relative to historical GS monotherapy (mPFS: 6.5 months *vs*. 5.36 months; mOS: 10.2 months *vs*. 9.43 months; DCR: 68.8% *vs*. 65%). However, given the limited sample size and nonrandomized design of this phase 1b/2 study, these comparisons remain exploratory but may nevertheless provide a potential clinical rationale for further investigations. Our in vitro cytotoxicity assays further supported this biological plausibility, demonstrating that expanded NK cells mediated potent, effector-to-target (E:T) ratio-dependent cytolytic activity against the K-562 and PANC1 cell lines (Supplementary Fig. 2e, f). Consistently, the clinical outcomes suggested a dose‒response relationship, with the highest NK cell dose (DL4) exhibiting an ORR of 60% (3/5), which was numerically greater than that of the low-dose cohorts, albeit with some response variability. Notably, our small-scale cohort unintentionally included a relatively high proportion of male patients with tumors located in the body and tail of the pancreas, which may limit the generalizability of our findings. Further validation through larger, randomized controlled trials with more representative populations is warranted. Considering the potential efficacy and favorable safety profiles, along with the fact that the MTD of NK cell infusion has yet to be reached, further investigations into higher cell doses are needed for the determination of the recommended phase 2 dose.

Certain special characteristics of patients warrant our attention. Specifically, P22 and P24, who were initially deemed unresectable, underwent conversion surgery after achieving satisfactory therapeutic responses. To date, there are limited reports, mainly case reports, on conversion surgery for MPC, with the resection rate still being less than 5%. These preliminary reports suggest that conversion surgery may prolong survival time.^33^ However, both P22 and P24 patients experienced recurrence following local resection, with relapse-free survival rates of 1.5 months and 4.1 months, respectively. A previous study reported that 75% of patients experienced recurrence after conversion surgery.^34^ This finding may serve as a warning that deciding whether and when to resect the tumor after achieving a satisfactory response requires more precise assessment criteria, such as monitoring minimal residual disease. ICI monotherapy has demonstrated limited efficacy in treating pancreatic cancer, with the clinical benefit largely confined to the rare microsatellite instability-high (MSI-H) subtype, which comprises less than 1% of cases.^35^ Notably, three patients (P2, P6, and P18), who received anti-PD-1 immunotherapy following NK cell combination therapy, exhibited relatively longer OS in our small cohort (27.0, 39.9 and 21.9 months, respectively; Supplementary Fig. 1a). Among them, P18 was a confirmed MSI-H case, whereas P2 and P6, although untested for MSI status, achieved a PR to prior NK cell-based therapy before subsequent PD-1 blockade. A similar clinical benefit was recently reported in a case study of metastatic pancreatic cancer patients treated with combined NK cell infusion and PD-1 blockade.^36^ We hypothesize that PD-1/PD-L1 blockade may directly reinvigorate the intratumoral NK cell response.^37,38^ Furthermore, our transcriptomic profiling revealed that NK cell combination therapy induces a proinflammatory, T-cell-activated microenvironment in responders, suggesting that this immune-remodeling niche may indirectly prime tumors for increased sensitivity to PD-1 inhibition. These findings indicate potential synergy between NK cell-based therapy and checkpoint blockade, supporting further investigations into combination strategies.

Another strength of our study is that, on the basis of our valuable treatment cohort, we used scRNA-seq and bulk TCR Vβ repertoire sequencing to characterize the immune features associated with patient responses with high-resolution insight. Although the limited cohort size imposed statistical constraints on the interpretation of immune dynamics, to our knowledge, this study remains the first comprehensive single-cell resolution atlas of immune response dynamics to NK cell-based therapy for PC. Here, we reannotated novel NK subtypes according to previous studies.^23–25,27^ The results revealed that c4-ZEB2, C5-IL7, c6-IL15, c10-NCR3, and c11-TNFSF8 accounted for an increased proportion of responders and overexpressed proinflammatory and effector molecules, including *IL7*, *IL15*, *IFNG*, *CD24*, and *NCR1*, implying an activating phenotype.^23,39,40^ However, the proportions of c0-KLRB1, c1-LAG3, and c2-IL6 in nonresponders increased, with genes such as *KLRC1*, *KLRB1*, *LAG3*, and *HAVCR2* showing relatively high expression levels but relatively low expression levels of genes associated with activation, indicating that those subsets manifested mainly exhausted and dysfunctional states,^23^ suggesting that further checkpoint blockade might be effective for nonresponders. Notably, these NKT cells exhibited an “adaptive NK”-like transcriptional pattern. Consistent with a previous study, researchers reported the expression of *CD3E/D/G* in adaptive NK cells, but they considered it an epigenetic alteration and thus defined these subsets as NK cells rather than NKT cells.^25^ Therefore, although this group of cells was named NKT cells in our study, they may still possess NK cell properties and need to be verified by flow cytometry in the future. A previous study revealed that APC patients who received NK cell infusion plus irreversible electroporation therapy presented an increased number of total NK cells after treatment; thus, these cells could be potential biomarkers.^17^ However, we found that circulating NK cells in APC patients are highly heterogeneous and that functional NK subtypes may be better efficacy predictors than total NK cells alone.

Myeloid cells play important roles in mediating NK cell-associated immunomodulation in cancer,^41,42^ and our analysis further confirmed that NK-myeloid cell crosstalk is a core mediator in response to NK cell-based therapy in PC. Intensive *TNFSF13*-*TNFRSF1A*, *FAS*-*FASLG*, *PVR*-*CD226*, *TNFRSF1A*-*GRN*, etc., were significantly different between NK cells and myeloid cells in responders, demonstrating an enhanced cytotoxic response of NK cells,^43,44^ which subsequently promoted clinical outcomes after NK cell infusions. However, nonresponders enriched more *NKG2A-* and *KIR*-based inhibitory receptor interactions between NK self-interaction or crosstalk with NKT cells, indicating a putative downregulation of NK cell cytotoxicity in nonresponsive patients. *NKG2A* and *KIR* inhibitory receptors are essential immune checkpoints on NK cells,^45,46^ suggesting that further combination treatment with anti-NKG2A antibodies or KIR antagonists may improve outcomes in nonresponders.

TCR diversity decreased over the course of consecutive NK cell-based treatments, suggesting potential tumor-specific clonal expansion in PC patients. Monitoring TCR clonotypic expansion is a feasible method to predict the clinical response to checkpoint inhibitors.^47^ Here, we observed that the efficacy of NK cell-based therapies in APCs could also be monitored. In addition, our data revealed that CD8^+^ Teff and CD4^+^ Tem extensions, but not CD8^+^ Tem and CD4^+^ Tcm extensions, were more likely to achieve favorable outcomes. Similarly, circulating CD8^+^ Teffs or CD4^+^ Tems were also found to be associated with a favorable response to ICI therapy in various tumor types.^48–51^ Notably, CD8^+^ Tems and CD4^+^ Tcms were thought to be associated with good prognosis in head and neck squamous cell carcinoma and gastric cancer patients^51,52^; however, these findings were related to unfavorable outcomes in PC patients. We attributed this to the functional heterogeneity of T-cell subtypes across distinct cancer types, as we further found that CD8^+^ Tcms and CD4^+^ Tcms expressed higher levels of the exhaustion marker gene *CTLA4* but lower levels of the integrin *ITGA4* and the chemokine receptor *CXCR5*, indicating their exhausted and inactivated status,^26^ and therefore, their responsiveness to treatment was reduced. We also found that expanded Tregs were associated with favorable outcomes in the Ti analysis (Fig. 5e), which may be due to the functional heterogeneity of Tregs responsive to the therapy. We found that posttreated Tregs presented increased proinflammatory immune scores, whereas baseline Tregs presented increased immunosuppressive function scores^53^ (Supplementary Fig. 7e). Nevertheless, we may not be able to exclude the causes related to the relatively low proportion of Tregs and the small sample size, and further investigations are needed.

Distinguishing infused NK cells from endogenous NK cells is essential for elucidating the mechanisms underlying NK cell-based therapy. However, unlike genetically modified cellular therapies,^54^ the unedited allogeneic NK cells used in this study lacked unique genetic tags, preventing direct tracking of the transfused population. Moreover, computational inference of the cellular origin on the basis of natural HLA mismatches was technically challenging owing to low coverage and high polymorphism of HLA genes in scRNA-seq data, compounded by ambient RNA noise. Despite these limitations, the observed expansion of functional NK and T-cell subsets in responders suggests that NK cell combination therapy may contribute to immune microenvironment reconstitution. This interpretation aligns with prior findings from adoptive cell immunotherapies, including CAR-T-cell trials, which have shown that successful cell transfer could remodel the tumor immune landscape by enhancing effector cell populations (e.g., T cells and NK cells) and attenuating immunosuppressive mechanisms (e.g., myeloid-derived suppressive cells).^55^ Although a direct causal link may not be established here, the correlation between immune subset expansion and clinical outcome implies that allogeneic NK cell infusion promotes a more responsive immunological state. Notably, four immune subsets, NK subsets c4-ZEB2 and c10-NCR3, along with CD4^+^ Tems and CD8^+^ Teffs, exhibited concordant expansion patterns in an independent cohort of patients receiving anti-PD-L1-based immunochemotherapy, despite differences in tumor type and treatment modality, suggesting their generalizable features of effective combination immunotherapy. However, the expansion of certain NK subsets (c5-IL7, c6-IL15, and c11-TNFSF8) was not observed in this external dataset, indicating a potential association specifically with NK-based therapy in pancreatic cancer and warranting further functional investigation. To unequivocally resolve NK cell origin, persistence, and mechanism, future studies could develop advanced computational algorithms for HLA-based cell assignment, alongside prelabeling strategies and in vivo murine models capable of longitudinal tracking of infused NK cells.

A primary weakness of this transcriptomic study is the small sample size. In addition, the absence of a control group receiving GS chemotherapy alone precludes a definitive attribution of the observed immunological differences between responders and nonresponders, specifically with respect to adoptive NK cell infusion. Although GS chemotherapy is associated with leukopenia,^30^ emerging evidence suggests that gemcitabine-based priming may modulate the TME by depleting immunosuppressive cells such as myeloid-derived suppressor cells (MDSCs) and Tregs while potentially enhancing peripheral IFNγ signaling.^56^ This immunomodulatory effect could ultimately synergize with subsequent ICIs or cellular immunotherapies.^56–58^ Elucidating the relative contributions of GS chemotherapy and adoptive NK cell transfer, as well as their potential synergistic effects on response-associated immune signatures, requires validation in future prospective randomized controlled trials or mechanistic studies in animal models.

Overall, this phase 1b/2 clinical trial of allogeneic NK cell therapy combined with GS in APC patients demonstrated its safety and preliminary efficacy, warranting further larger, prospective, randomized controlled trials. Allogeneic NK cells in combination with other established regimens, such as FOLFIRINOX or gemcitabine/nab-paclitaxel, may broaden the global therapeutic applicability. Additionally, our study identified potential clinical outcomes relevant to circulating NK and T-cell subsets in APCs, providing novel insight into the dynamic transcriptional underpinnings of the peripheral immune landscape with distinct responses to NK cell-based therapy. These comprehensive dynamic transcriptomes and TCR sequences provide valuable resources for deciphering the functional states and dynamics of immune cells after NK cell-based therapy in other solid tumors.

## Materials and methods

### Ethic statements

The protocol was approved by the Beijing Hospital Institutional Review Board (2018BJYYEC-245) and was conducted in accordance with the Declaration of Helsinki and Good Practice guidelines of the International Conference on Harmonization. All patients provided written informed consent.

### Study design and interventions

This first-in-human, single-arm, nonrandomized, phase 1b/2 clinical trial was conducted at Beijing Hospital (China) to evaluate the safety and feasibility of allogeneic NK cell administration with GS chemotherapy in APC patients in a dose-escalation design (ChiCTR1900021764). Adult patients with APC who were scheduled to receive GS as first-line systemic therapy were recruited from outpatient clinics by treating physicians. Individuals were assigned to four dose-level cohorts: 1 × 10^9^ NK cells as an initial dose, 2 × 10^9^ NK cells, 4 × 10^9^ NK cells, and 8 × 10^9^ NK cells. The sample size was dependent upon the observed safety profile within the modified dose-escalation design. Potential bias was minimized through matching the demographic characteristics. The study was initiated by the investigators.

Each 21-day treatment cycle consisted of standard GS chemotherapy combined with NK cell administration during inpatient care by physicians. Patients received gemcitabine on days 1 and 8 (1,000 mg/m^2^ intravenously over 30 minutes), along with oral S-1 twice daily from day 1 to day 14. The daily S-1 dose was determined by body surface area: 60 mg for <1.25 m², 80 mg for 1.25 to 1.5 m², and 100 mg for > 1.5 m².^30^ The expanded NK cell products were infused intravenously between days 14 and 21 following chemotherapy, aiming to minimize the potential hematological toxicity to the infused NK cells. NK cells were administered once per cycle in the lower-dose cohorts (1 × 10^9^ cells, 2 × 10^9^ cells, and 4 × 10^9^ cells), whereas the highest-dose cohort (8 × 10^9^ cells) received half the total dose fractionated over two consecutive days to mitigate risks associated with large-volume infusion. Patients were scheduled to receive 6–8 cycles of GS chemotherapy followed by S-1 monotherapy maintenance. Local treatments were permitted throughout. NK cell infusion was combined with the first four cycles of GS chemotherapy, unless unacceptable adverse effects, disease progression, or patient withdrawal occurred. Patients who expressed a strong desire to continue NK cell therapy beyond four cycles could receive compassionate treatments, with the treatment cycle frequency determined by the investigators on the basis of individual tolerance and response. Participant visits were followed for two longitudinal time points per cycle (before gemcitabine administration and after NK cell infusion). Computed tomography (CT) scanning or magnetic resonance imaging (MRI) was performed at baseline and every two cycles of treatment thereafter until disease progression. Clinical research coordinators were set up to guarantee subjects’ compliance.

### Endpoints and assessments

The primary endpoint was to evaluate safety. The reporting of AEs starts with the receipt of GS chemotherapy and continues through the clinical trial. AEs potentially related to GS chemotherapy or NK cell infusion were recorded according to the National Cancer Institute Common Terminology Criteria for Adverse Events, version 5.0 (NCI CTCAE v.5.0). The chemotherapy-related AEs were judged by the physicians on the basis of the timing of onset, as well as the known toxicity profiles of the chemotherapeutic agents. The dose-limiting toxicity of NK cell therapy was defined as follows: (i) Hematologic toxicity: Grade 4 neutropenia lasting more than 7 days; febrile neutropenia; neutropenic infection of Grade 3 or higher; thrombocytopenia of Grade 3 or higher with bleeding; Grade 4 thrombocytopenia; and Grade 4 anemia. (ii) Nonhematologic toxicity: Grade 3 or higher nausea, vomiting or diarrhea despite maximal supportive care; any other clinically significant nonhematologic toxicity of Grade 3 or higher (excluding asymptomatic biochemical abnormalities that are clinically insignificant and resolve to Grade 2 or lower within 7 days); any grade DLT based on the investigator’s discretion.

The second endpoints were the response rate and survival. Clinical responses were assessed by CT scanning or MRI at baseline and every two cycles until progressive disease (PD), according to RECIST 1.1. At least two sequential CT scans were required to confirm PR or SD. Single-site tumor progression or new lesions were recorded as PD. For long-term survival, overall survival (OS) was defined as the time from enrollment to the follow-up deadline or the date of death from any cause. Progression-free survival (PFS) was measured from enrollment to the follow-up deadline or the date of progression confirmed by the investigator’s assessment. Subgroup analyses were conducted in patients with metastatic pancreatic cancer and at distinct dose levels. Missing data for any of the outcomes were deleted during the statistical process.

The exploration endpoint was to estimate the dynamic peripheral immune landscape in response to this therapeutic strategy in the APC.

### Allogeneic NK cell manufacturing and release criteria

Peripheral blood (PB) was obtained from direct relatives of patients, and cord blood (CB) was collected from fetuses at delivery at Beijing Hospital. Mononuclear cells were isolated via density gradient centrifugation via Lymphocyte Separation Medium (Tianjin Haoyang Biological Manufacture Co., Ltd. Tianjin, China) under GMP-compliant conditions. NK cells were activated and expanded over approximately two weeks according to the manufacturer’s protocol for an ex vivo NK Cell Expansion Kit (Beijing Wukang Xinxing Technology Co., Ltd., Beijing, China), with media containing heat-inactivated autologous plasma and recombinant human interleukin-2 (Beijing SL Pharmaceutical Co., Ltd., Beijing, China). The cells were harvested on days 14 to 16, washed, and resuspended in 100 mL of saline-based solution containing 0.6% human serum albumin (albumin, Baxalta Pharmaceutical Company, USA) for immediate intravenous administration to patients.

Quality control included sterility testing via the BACTEC™ Blood Culture System (BD Diagnostics, Sparks, MD, USA), mycoplasma detection via quantitative real-time PCR-based kits (TransGen Biotech, Beijing, China), endotoxin assessment via the LAL assay (Xiamen Bioendo Technology Co., LTD, Xiamen, China), and viability measurement via ViaStain™ AOPI staining (Revvity, Waltham, MA, USA). The purity of CD3⁻CD56⁺ NK cells was determined via flow cytometry (BD FACSCanto II). The following release criteria were used: sterility and mycoplasma negativity, endotoxin concentration < 0.25 EU/mL, viability ≥ 85%, and CD3^-^CD56^+^ NK cell purity ≥ 70%.

Cytotoxic activity was evaluated against the K-562 and PANC-1 cell lines via a time-resolved fluorescence-based cytotoxicity assay (DELFIA®; Revvity). Specific lysis was calculated after coculture at various E:T ratios. Products that achieved ≥ 40% specific lysis of K-562 cells at an E:T ratio of 2.5:1 were released from the infusion.

### scRNA-seq data processing

PBMCs were isolated by density gradient centrifugation. Single-cell suspensions were processed using the 10 × Genomics Chromium platform (performed by CapitalBio Technology, Beijing, China) to generate gel beads-in-emulsion (GEMs) following the manufacturer’s protocol. Raw sequencing data were processed with Cell Ranger (v.6.0.1) for demultiplexing, alignment to the GRCh38 reference genome, and generation of a unique molecular identifier (UMI) count matrix. Potential doublets were identified and removed using Scrublet (v.0.2.1). Subsequent analysis was performed with Seurat (v.4.3.0). Cells were filtered based on the following quality thresholds: 500–30,000 UMIs, 500–6,000 genes detected, and mitochondrial/erythrocyte gene content below 15% and 5%, respectively, to retain high-quality cells for downstream analysis.

### Dimension reduction and unsupervised clustering for scRNA-seq data

Unsupervised clustering was performed with Seurat (v.4.3.0). The top 2,000 highly variable genes were selected for principal component analysis (PCA). Next, nonlinear dimensionality reduction was performed using uniform manifold approximation and projection (UMAP) and t-distributed stochastic neighbor embedding (t-SNE) with the top 50 principal components, followed by graph-based clustering. Cell clusters were annotated according to canonical markers^21,22^ and differentially expressed genes (min.pct = 0.1, logfc.threshold = 0.25). The detailed list of annotation markers for each cell subtype is available in the Supplementary Materials and methods.

### Pathway analysis

DEGs with |log2 fold-change| > 0.5 and adjusted *p*-value < 0.05, unless otherwise noted in figure legends, among groups were detected by the *FindMarkers* function. GO and KEGG enrichment analysis on DEGs in this study were performed by R package clusterProfiler (v.4.8.1). GSEA was conducted in the Omicshare platform (https://www.omicshare.com/), based on the hallmark or C5 GO biological process (GO-BP) gene sets from the Molecular Signatures Database.

### Definition of functional gene sets and calculation of signature score

To assess the functional heterogeneity of NK cell subpopulations, we defined active, mature, adaptive, and HLA-related gene sets identified in previous studies.^25,27^ To evaluate posttreatment T cell dynamics, we employed inhibitory receptor gene sets and integrins gene sets identified in a previous study.^26^ Each enrichment score of a specific gene set was calculated using the *AddModuleScore* function. The detailed lists of genes included in each signature are provided in the Supplementary Materials and methods.

### Application of predictive index and therapeutic index

We applied the modified predictive index (Pi) and therapeutic index (Ti)^26^ to evaluate associations between immune cell subsets and clinical response. Pi measures the correlation between baseline cellular proportions with the initial tumor size, while Ti measures the correlation between the post-treatment cellular proportion with tumor size changes. A positive Pi or Ti indicates that a higher baseline or posttreatment cell level is associated with a smaller initial tumor or greater tumor shrinkage following treatment, thus predicting or mediating a favorable response, respectively. Pi analysis included all pre-treatment samples (*n* = 7). Ti analysis was derived from patients with paired samples (*n* = 4: P22-post3, P24-post1, P26-post3, P27-post1), excluding P20 who lacked a CT scan at progression. Tumor size was the maximum long diameter; shrinkage was the percent change from baseline (Supplemental Tables S5, 6).

### Cell-cell interaction analysis

We used CellPhoneDB (v.2.1.5) to infer cell-cell interactions between NK/NKT cells and other immune cells in this study. This method infers the potential interaction counts between two cell subsets based on gene expression level and provides the significance through a permutation test (1000 times). We extracted significant ligand-receptor pairs with *p*-value < 0.05. R package ktplots (v.1.2.5) was used for visualization.

### Analysis of public scRNA-seq dataset

The public scRNA-seq dataset was obtained from the Gene Expression Omnibus (GEO) database with the accession number GSE266919.^28^ The public data were then processed, normalized, scaled, and clustered using Seurat (v.4.3.0). T and NK cell subsets were defined by integrating the original study’s annotations with the expression patterns of characteristic marker genes identified in our data. Finally, we compared the proportional changes of T/NK cell subsets between responders and nonresponders after treatment, focusing on subsets that showed consistent expansion patterns across both our dataset and this validation cohort.

### Bulk RNA isolation and TCR repertoire sequencing

Separated PBMCs were stored in RNAlater (Thermo Fisher Scientific) and conserved at 4 °C until RNA extraction. The procedure of RNA isolation and amplification, and TCR repertoire sequencing was performed at Chengdu ExAb Biotechnology Ltd. at China. Sequences were processed and analyzed based on MiXCR (v.3.0.13) tool. Subsequent diversity and clonotype distribution analysis were performed using R package immunarch (v.0.9.0).

### Statistical analysis

Descriptive statistics for continuous variables include means with standard deviations or medians with minima and maxima. Categorical variables were summarized via counts and percentages, and 95% confidence intervals were calculated via the Clopper‒Pearson exact method. Survival analysis was performed via the Kaplan‒Meier method, and 95% confidence intervals were calculated for medians and curves. Statistical analysis was performed via IBM SPSS software (SPSS Inc., v.27.0), GraphPad Prism 8 (GraphPad Software, Inc.), R (v.4.2.0) and the OmicShare platform (https://www.omicshare.com/). Statistical significance in our study was determined via the Wilcoxon test or two-tailed Student’s *t* test, as described in the figure legends. A *p* value < 0.05 was considered statistically significant. All boxplots presented show the median, interquartile range, and minima/maxima of the samples. The data in the bar graphs were plotted as the means ± SEMs.

## Supplementary information

Supplementary Materials

Trial Protocol

## Data Availability

The raw scRNA-seq and bulk TCR Vβ sequencing data generated during the current study are deposited in the Genome Sequence Archive (GSA) database (https://bigd.big.ac.cn/gsa) under accession numbers HRA008574 and HRA009425. No new algorithms were developed in this study, and all the data and materials supporting the findings in this study are available from the corresponding author upon reasonable request.
