# Supplementary material for: Dynamic transcriptional immune landscape in response to NK-cell therapy combined with gemcitabine plus S-1 in advanced pancreatic cancer: a phase 1b/2 trial
Source: Signal Transduct Target Ther. 2025 Nov 21;10:381. doi: 10.1038/s41392-025-02488-1 (PMC12635223; doi:10.1038/s41392-025-02488-1)
Supplement: Supplementary file 1 — Supplementary Materials [file 41392_2025_2488_MOESM1_ESM.docx]

Supplementary Materials for

**Dynamic transcriptional immune landscape in response to NK cell therapy combined with gemcitabine plus S-1 in advanced pancreatic cancer: a phase 1b/2 trial**

Qin Tan^1#^, Yifei Li^2#^, Caixia Liu^1^, Jing Xu^1^, Jinlian Tong^1^, Jiangyong Yu^1^, Yingying Huang^3^, Xueqing Hu^3^, Sen Qin^1^, Fei Xiao^2*^, Yunbo Zhao^3*^, Jie Ma^1*^

Correspondence to: [majie4685@bjhmoh.cn](mailto:majie4685@bjhmoh.cn) (J.M.); [Zhaoyb1206@163.com](mailto:Zhaoyb1206@163.com) (Y.Z.); [xiaofei3965@bjhmoh.cn](mailto:xiaofei3965@bjhmoh.cn) (F.X.)

**This PDF file includes:**

Materials and methods

Figures. S1 to S7

Tables. S1 to S6

Materials and methods

Allogeneic NK cell manufacturing and release criteria

Peripheral blood (PB) was obtained from each patient’s direct relatives, and cord blood (CB) was collected from fetuses at delivery at Beijing Hospital. Under good manufacturing practice (GMP)-compliant laboratory conditions, mononuclear cells from PB or CB were separated via density gradient centrifugation via Lymphocyte Separation Media (Tianjin Haoyang Biological Manufacture Co., Ltd. Tianjin, China). Subsequently, the NK cells were activated and expanded for approximately 2 weeks according to the manufacturer’s protocol of the *ex vivo* NK cell Expansion Kit (Beijing Wukang Xinxing Technology Co. Ltd., Beijing, China). Briefly, mononuclear cells were resuspended in working solution 1 containing 10% heat-inactivated autologous plasma and then transferred to cell-culture flasks. On day 4, working solution 3 (mixed in a 1:1 ratio with working solution 1 and solution 2) containing 5% autologous plasma was added. On day 6, the cultured cells were transferred to CultiLife® Evabags (Takara Bio) and supplemented with working solution 4 (containing 0.6% autologous plasma and recombinant human interleukin-2 (Beijing SL Pharmaceutical Co., Ltd., Beijing, China)). The cells were then expanded by adding working solution 4 every 2 or 3 days. On days 14 to 16, cells were harvested, washed, and resuspended in 100 mL of saline-based solution containing 0.6% human serum albumins (albumin, Baxalta Pharmaceutical Company, USA) for immediate intravenous administration to patients.

Quality control tests were performed by assessing samples taken during the culture period and the final product. For sterility testing, aliquots of cultured samples were aseptically inoculated into BACTEC™ Lytic/10 Anaerobic/F, BACTEC™ Plus Aerobic/F, and BACTEC™ Myco/F Lytic culture vials, and continuously monitored on the BACTEC™ FX automated microbial detection system (BD Diagnostics, Sparks, MD, USA). Bacterial growth till approximately day 7 should be reported as positive. The Mycoplasma contamination was assessed using MagicPure® Mycoplasma DNA Kit and TransDetect® qPCR Mycoplasma Detection Kit (TransGen Biotech, Beijing, China). The endotoxin was tested using the Limulus Amebocyte Lysate (LAL) test (Xiamen Bioendo Technology Co., LTD, Xiamen, China). The viability of expanded NK cells was counted using the Cellometer K2 Fluorescent Cell Counter and ViaStain™ AOPI Staining Solution (Revvity, Waltham, MA, USA). The purity of CD3^-^CD56^+^ NK cells in the end-product was determined by flow cytometry (BD FACSCanto II) using mouse anti-human CD3-APC, CD56-PerCP-Cy5.5, and CD16-FITC (BD Biosciences, CA, USA). Final NK cell product release criteria included: sterility and mycoplasma testing negative, endotoxin assay ＜ 0.25 EU/ml, cell viability ≥ 85%, and purity of CD3^-^CD56^+^ NK cells ≥ 70%.

*In vitro* cytotoxicity assay

Expanded NK-cell (end-products) cytotoxicity against the K-562 or PANC-1 as target cells was measured with the DELFIA® TRF (time-resolved fluorescence) Cytotoxicity Kit (Revvity, Waltham, MA, USA) according to the manufacturer’s instructions. Briefly, target cells were labeled with ELFIA BATDA reagent for 10 minutes at 37℃, washed, and plated at 1 × 10^4^ cells per well in 96-well tissue culture plates. NK cells were added at various effector-to-target (E: T) ratios ranging from 80:1 to 2.5:1, and incubated for 1 hour (K-562) or 2 hours (PANC-1) at 37℃ under 5% CO_2_. Supernatants were mixed with DELFIA Europium solution for 15 minutes at room temperature. The Europium signal was measured on the Varioskan LUX multimode microplate reader (Thermo Fisher Scientific, Waltham, MA, USA). Specific cytotoxicity was calculated as follows: % cytotoxicity = 100 × (experimental release - spontaneous release) / (maximum release – spontaneous release). Products achieving ≥ 40% specific lysis of K-562 targets at an E:T ratio of 2.5:1 were released from the infusion.

Peripheral blood sample collection

Fresh PB samples were collected at baseline and multiple timepoints across clinical trial cycles in ethylenediaminetetraacetic acid anticoagulant tubes. According to previous studies, patients with the best overall response (BOR) of complete response (CR) and partial response (PR) were classified as responders, while patients with stable disease (SD) or progressive disease (PD) were classified as nonresponders^1^. A total of seven patients were enrolled and 19 PB samples were included for scRNA-seq, including responders (R, n=4, including PR subjects P22, P24, P26, and 27) and nonresponders (NR, n = 3, including SD/PD subjects P16, P20, and P21). We also performed bulk TCR Vβ repertoire sequencing for 17 PB samples from three responders (P24, P26, and P27).

scRNA-seq data generation and processing

PBMCs were freshly isolated using density gradient centrifugation, and then a single-cell suspension was loaded onto the Chromium single-cell controller (10× Genomics) to generate single-cell gel beads in the emulsion according to the manufacturer’s instructions. In brief, single cells were resuspended in phosphate-buffered saline containing 0.04% bovine serum albumin. Approximately 1.3 × 10^4^ cells were added to each channel, and the target cell that will be recovered was estimated to be about 8,000 cells. Captured cells were lysed and the released mRNA was barcoded through reverse transcription in individual nanoliter-scale Gel bead in emulsion, then reverse transcribed in aS1000TM Touch Thermal Cycler (Bio Rad) at programmed at 53℃ for 45 min, 85℃for 5 min, and then held at 4℃. The cDNA was generated and then amplified, and quality-assessed using the Agilent 4200 system. scRNA-seq libraries were constructed using the Single cell 3′ Library and Gel Bead Kit V3.1 according to the manufacturer’s instructions. The libraries were finally sequenced using the Illumina NovaSeq 6000 sequencer with a sequencing depth of at least 1 × 10^5^ reads per cell with 150 bp paired-end reads (performed by CapitalBio Technology, Beijing, China).

The Cell Ranger (v.6.0.1) provided by 10× Genomics was applied to aggregate raw data, filter low-quality reads, align reads to GRCh38 human reference genome, assign cell barcodes, and generate the unique molecular identifier (UMI) matrix. To exclude data from droplets containing more than one cell, doublet detection and removal were performed using Scrublet (v.0.2.1) with default parameters. R library Seurat (v.4.3.0), was used for analyzing the scRNA-seq data. We further quantified the number of genes and UMI counts for each cell, and kept high-quality cells with thresholds of 500–30,000 UMIs, 500–6,000 genes, less than 15% mitochondrial gene counts, and less than 5% erythrocyte gene counts, to ensure that most of the heterogeneous cell types were included for downstream analysis.

Dimension reduction and unsupervised clustering for scRNA-seq data

Unsupervised clustering was performed according to the standard workflow in R package Seurat with default parameters, unless otherwise indicated. By default, we first identified the 2,000 highly variable genes within each dataset, and then generated a combined gene set after removal of unique genes from each dataset. Principal component analysis (PCA) was performed on the variable gene matrix to reduce noise using the *RunPCA* function, and the top 50 principal components were calculated using the *FindNeighbors* function for downstream analysis. We further used the *RunHarmony* function to correct the batch effects from different samples. Then, the resolution parameters of the *FindClusters* function were different for distinct cell types, with 0.7 for all immune cells, 0.9 for NK cells, and 1.3 for T cells. Uniform manifold approximation and projection (UMAP) t-distributed stochastic neighbor embedding (t-SNE) were performed for visualization. Clusters were then classified and annotated based on expressions of canonical markers^2,3^ and marker genes found using the *FindAllMarkers* function with the parameter “min.pct = 0.1, logfc.threshold = 0.25” of particular cell types.

Major immune cell lineages include lymphoid lineage (T/NK cells: *CD3D*, *CD3E*, *NKG7* and *NCAM1*; B cells: *CD79A*, *CD79B*, *MS4A1*, *MZB1* and *XBP1*), myeloid lineage (neutrophils: *FCGR3B*, *CSF3R*, *S100A8* and *S100A9*; CD14 monocytes: *CD14*, *CST3*, *LYZ* and *MS4A7*; CD16 monocytes: *FCGR3A*, *CST3*, *LYZ* and *MS4A7*; mDC: *CD1C* and *ITGAX*; pDC: *ITM2C* and *LILRA4*), Megakaryocyte (*PPBP* and *PF4*) and hematopoietic stem and progenitor cells (HSPCs: *CD34*).

In T cell lineage, we finally annotated 4 subsets of CD4^+^ T cells including central memory (CD4^+^ Tcm: *CD3D*, *CD3G*, *CD4*, *GPR183,* and *S100A4*), effector memory (CD4^+^ Tem: *CD3D*, *CD3G*, *CD4*, *GPR183*, *S100A4*, *GZMA,* and *GZMK*), naïve (CD4^+^ Tn: *CD3D*, *CD3G*, *CD4*, *SELL,* and *CCR7*) and regulatory (Treg: *CD3D*, *CD3G*, *CD4*, *FOXP3,* and *IL2RA*); 3 subsets of CD8^+^T cells including effector (CD8^+^ Teff: *CD3D*, CD*3G*, *CD8A*, *CD8B*, *GZMA,* and *GNLY*), effector memory (CD8^+^ Tem: *CD3D*, *CD3G*, *CD8A*, *CD8B*, *GPR183*, *S100A4*, *GZMA,* and *GZMK*) and naïve (CD8^+^ Tn: *CD3D*, *CD3G*, *CD8A*, *CD8B,* and *LEF1*); 1 subtypes of double-positive T cells (DPT: *CD3D*, *CD3G*, *CD4*, *CD8A,* and *CD8B*), 1 subtypes of proliferating T cells (Tprof: *CD3D*, CD3G, *MKI67,* and *STMN1*) and 1 subtypes of NK/NKT cells (*NCAM1* and *FCGR3A*). We identified NK cell clusters by their entire signature genes and named them by selecting one specific signature gene after considering the observed expression pattern.

Pathway analysis

Differentially expressed genes (DEGs) with |log2 fold-change| > 0.5 and adjusted *P*-value < 0.05, unless otherwise noted in figure legends, among groups were detected by the *FindMarkers* function. GO and KEGG enrichment analysis on DEGs in this study were performed by R package clusterProfiler (v.4.8.1). GSEA was conducted in the Omicshare platform (https://www.omicshare.com/), based on the hallmark or C5 GO biological process (GO-BP) gene sets from the Molecular Signatures Database.

Definition of functional gene sets and calculation of signature score

To estimate the function variation of NK cell subpopulations, we defined active, mature, adaptive, and HLA-related gene sets identified in previous studies^4,5^. The active-like NK cell gene set was defined as *CD69*, *NR4A2*, *DUSP1*, *FOS*, *JUN*, *JUNB,* and *PPP1R15A*. The mature-like NK cell gene set was defined as *SPON2*, *TBX21*, *B3GAT1*, *GZMA*, *GZMH*, *GZMB*, *PRF1*, *ARPC2*, *CFL1*, *CST7,* and *ACTB*. The adaptive-like NK cell gene set was defined as *KLRC2*, *CD52*, *CCL5*, *IL32,* and *HLA-DPB1*. The HLA-like NK cell gene set was defined as *HLA-DRA* and *CD74*. To evaluate the transcriptional dynamics of T cells in patients after treatment, we employed inhibitory receptor gene sets and integrins gene sets identified in a previous study^1^. The inhibitory receptor gene set was defined as *CD244*, *CD160*, *CTLA4*, *PDCD1*, *TIGIT*, *LAYN*, *LAG3,* and *HAVCR2*, and the integrins gene set was defined as *ITGB1*, *ITGA4*, *ITGA1*, *ITGAL*, *ITGB2*, *ITGAE*, *ITGAV*, *ITGA6*, *ITGA5*, *ITGA2*, and *ITGB7*. To estimate the difference of Treg transcriptional functions after treatment, we defined the Treg immunosuppressive score and Treg proinflammatory score after a comprehensive compilation of previous studies. The Treg immunosuppressive score was defined as *IL1R1*, *IL21R*, *TNFRSF9*, *REL*, *CTLA4,* and *IL2RA*^6^. The Treg proinflammatory score was defined as *IFNG*, *GZMK*, *GZMA*, *GZMH*, *GZMB,* and *PRF1*^7-9^. Each enrichment score of a specific gene set was calculated using the *AddModuleScore* function.

Application of predictive index and therapeutic index

To avoid the sample-size limitation, we employed the modified predictive index (Pi) and the therapeutic index (Ti) to systematically investigate the association of distinct cell subtypes with clinical responses, as previously reported^1^, The Pi measures the correlation between baseline cellular proportions with the initial tumor size, while the Ti measures the correlation between the post-treatment cellular proportion with tumor size changes. A positive Pi or Ti represents that a higher level of baseline or post-treatment cellular proportion of the corresponding immune cell subtype is associated with a smaller tumor size or higher degree of tumor shrinkage following treatment, thus predicting or mediating a favorable response, respectively. A negative Pi or Ti represents that the corresponding immune cell subtype predicts or mediates an unfavorable response, respectively. Notably, Pi was derived from patients with pre-treatment samples to measure the baseline level of immune cell proportions (n = 7, all pre-treatment samples). While the Ti was applied to calculate the relative dynamic alteration of immune cell proportions, and thus it was derived from patients with paired blood samples (n = 4, P22-post3, P24-post1, P26-post3, and P27-post1, we excluded P20 in Ti analysis, who was peritoneal metastatic but with no abdominal CT scan when reached PD. The tumor size was measured by the maximum tumor long diameter, and the tumor shrinkage was calculated by the percentage increase/decrease in tumor size after treatment (Supplemental Table S5, 6).

Cell-cell interaction analysis

We used CellPhoneDB (v.2.1.5) to infer cell-cell interactions between NK/NKT cells and other immune cells in this study. This method infers the potential interaction counts between two cell subsets based on gene expression level and provides the significance through a permutation test (1000 times). We extracted significant ligand-receptor pairs with *P*-value < 0.05. R package ktplots (v.1.2.5) was used for visualization.

Survival analysis

To assess the relationship between the proportion of specific cell subsets after treatment and overall survival, the survival curves were performed by the Kaplan–Meier method with R package survival (v.5.5.5), and visualized using the ‘*ggsurvplot*’ function of the survminer (v.0.4.9) package. *P*-value was assessed by the log-rank test statistic between two groups.

Analysis of public scRNA-seq dataset

The public scRNA-seq dataset was downloaded from the National Center for Biotechnology Information (NCBI)’s Gene Expression Omnibus (GEO) with the accession number GSE266919^10^. For public scRNA-seq dataset analysis, the R package Seurat (v.4.3.0) was used. First, raw counts were normalized and scaled, and the top 2,000 highly variable genes were selected for PCA. Next, Nonlinear dimensionality reduction was performed using UMAP with the top 50 principal components, followed by graph-based clustering. We then defined T/NK cell subsets by integrating the cell type annotation results from the original public dataset with the expression patterns of characteristic marker genes identified in our data. Finally, we compared the proportional changes of T/NK cell subsets between responders and nonresponders post-treatment, focusing on subsets that showed consistent expansion patterns across both our dataset and this validation cohort.

Bulk RNA isolation and TCR repertoire sequencing

PBMC suspension was obtained by using density gradient centrifugation with lymphocyte separation medium (TianJin HaoYang Biological Manufacture Co., Ltd., Tianjin, China), then stored in RNAlater (Thermo Fisher Scientific) and conserved at 4℃ until RNA extraction. The procedure of RNA isolation and amplification, and TCR repertoire sequencing was performed at Chengdu ExAb Biotechnology Ltd. at China. Sequences were processed and analyzed based on MiXCR (v.3.0.13) tool. Each unique complementarity determine region 3 (CDR3) of T cell receptor beta chain (TRB) gene which was defined as a clone, and cells with the same CDR3 sequences were identified as the same clonotype. Subsequent diversity and clonotype distribution analysis were performed using R package immunarch (v.0.9.0).

Statistical analysis

Descriptive statistics for continuous variables include means with standard deviations or medians with minima and maxima. Categorical variables were summarized via counts and percentages, and 95% confidence intervals were calculated via the Clopper‒Pearson exact method. Survival analysis was performed via the Kaplan‒Meier method, and 95% confidence intervals were calculated for medians and curves. Statistical analysis was performed via IBM SPSS software (SPSS Inc., v.27.0), GraphPad Prism 8 (GraphPad Software, Inc.), R (v.4.2.0) and the OmicShare platform (https://www.omicshare.com/). Statistical significance in our study was determined via the Wilcoxon test or two-tailed Student’s *t* test, as described in the figure legends. A *P* value < 0.05 was considered statistically significant. All boxplots presented show the median, interquartile range, and minima/maxima of the samples. The data in the bar graphs were plotted as the means ± SEMs.

Figure. S1.

**
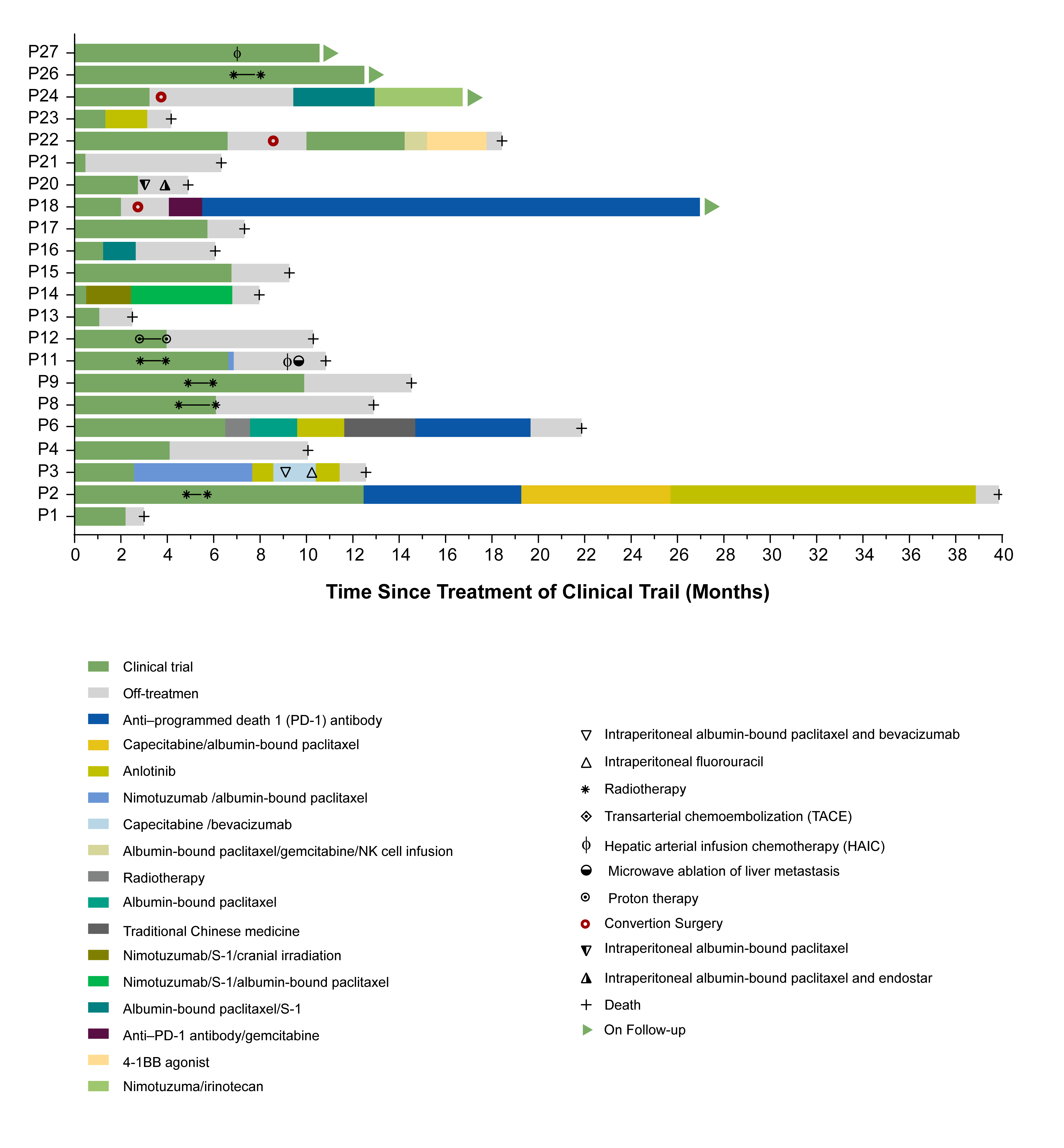
**

**Figure. S1. Therapeutic regimens (n=22).** Swimmer’s plot depicting the time on study, subsequent therapies, and present status for all enrolled patients, excluding three patients with no follow-up data (P10, P19, and P25). Green arrows indicate ongoing survival, and black “+” denotes patient death.

Figure. S2.

**
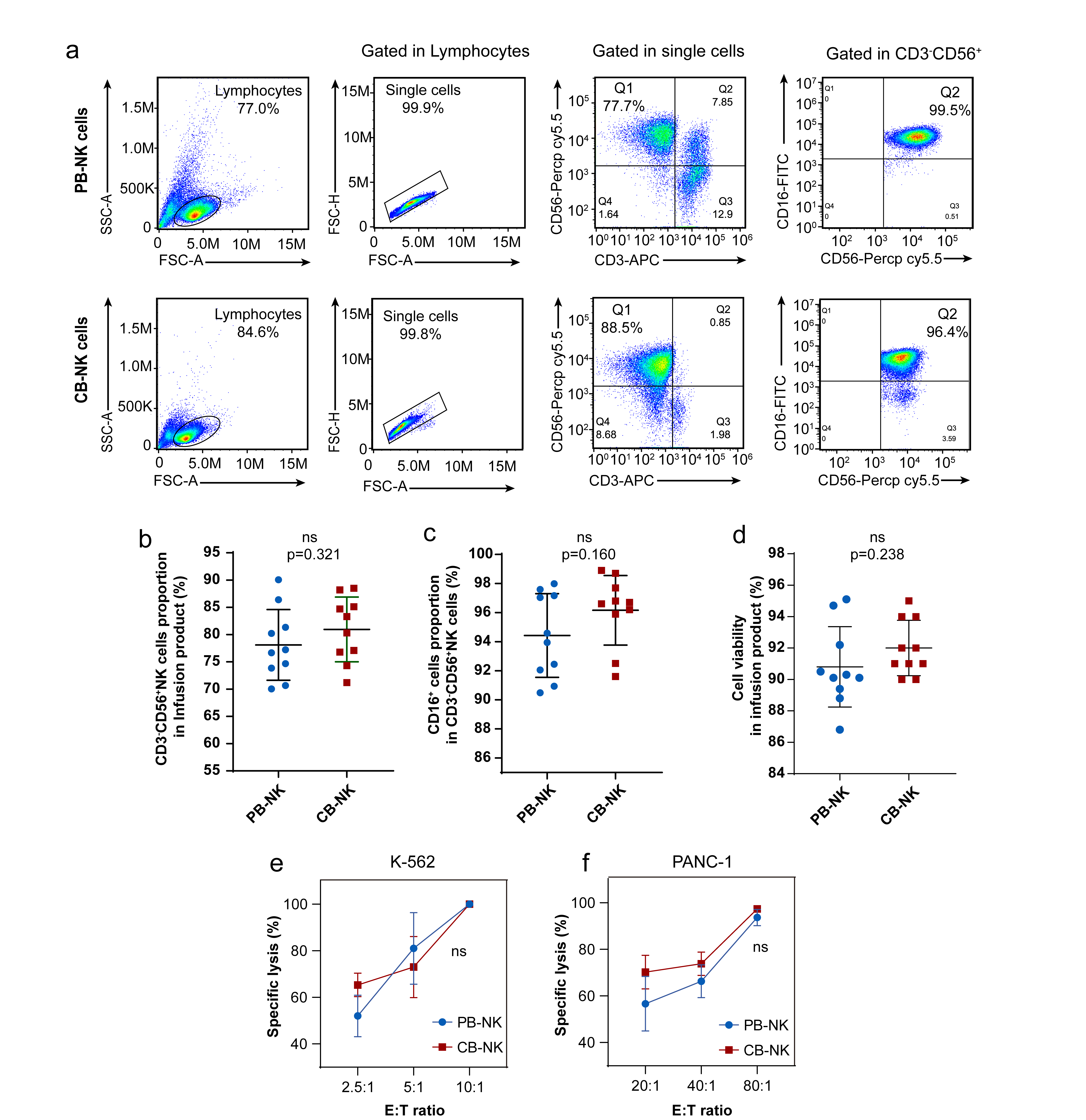
**

**Figure. S2. Characterizations of expanded allogeneic NK cell products.** **a** Representative flow cytometry dot plots for NK cell phenotypes. **b** Purity of CD3^-^CD56^+^ NK cells in infusion products from peripheral blood (PB) or cord blood (CB); Each point represents one sample (n = 10 per group). **c** Proportion of CD16^+^ cells gated in CD3^-^CD56^+^ NK cells (n = 10 per group). **d** Cell viability of infused NK cell products from peripheral blood (PB) or cord blood (CB) (n = 10 per group). **e** NK cell cytotoxicity against K-562 target cells at 2.5:1, 5:1 and 10:1 effector to target (E:T) ratios. **f** cytotoxicity of NK cells against PANC-1 target cells at 20:1, 40:1 and 80:1 E:T ratios. Data are represented as mean ± SD.

Figure. S3.


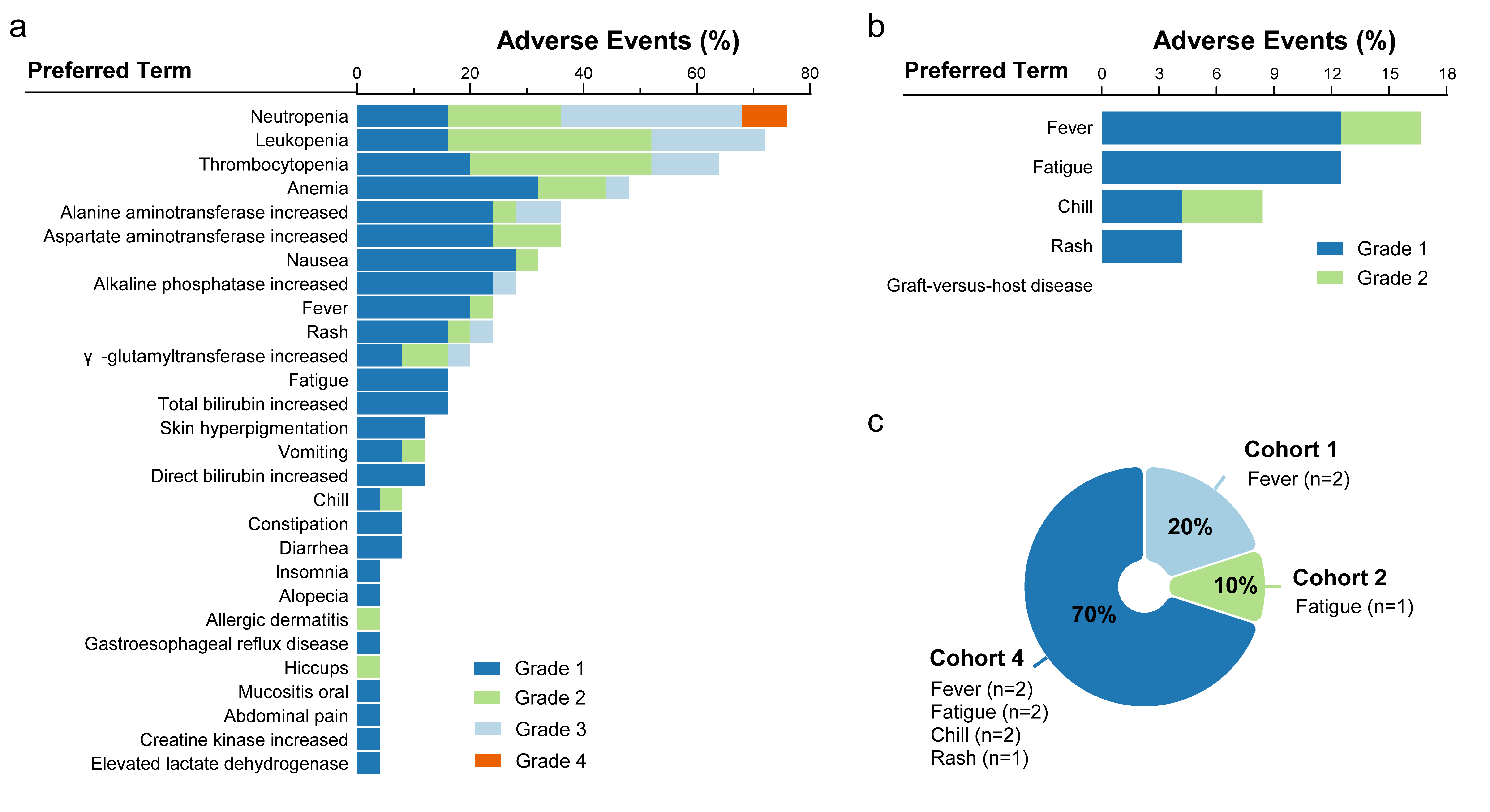


**Figure. S3. Treatment-related adverse events (TRAEs). a** TRAEs occurring in patients of each grade, attributable to the combination treatment (allogeneic NK cell infusion combined Gemcitabine plus S-1 (GS) chemotherapy). All enrolled APC patients (n = 25) are included. **b** TRAEs possibly occurring in patients with NK cell therapy (n = 24), excluding patient P25 who dropped out due to severe chemotherapy-related toxicity. **c** Pie plot showing NK cell infusion-related adverse events in each cohort.

Figure. S4.


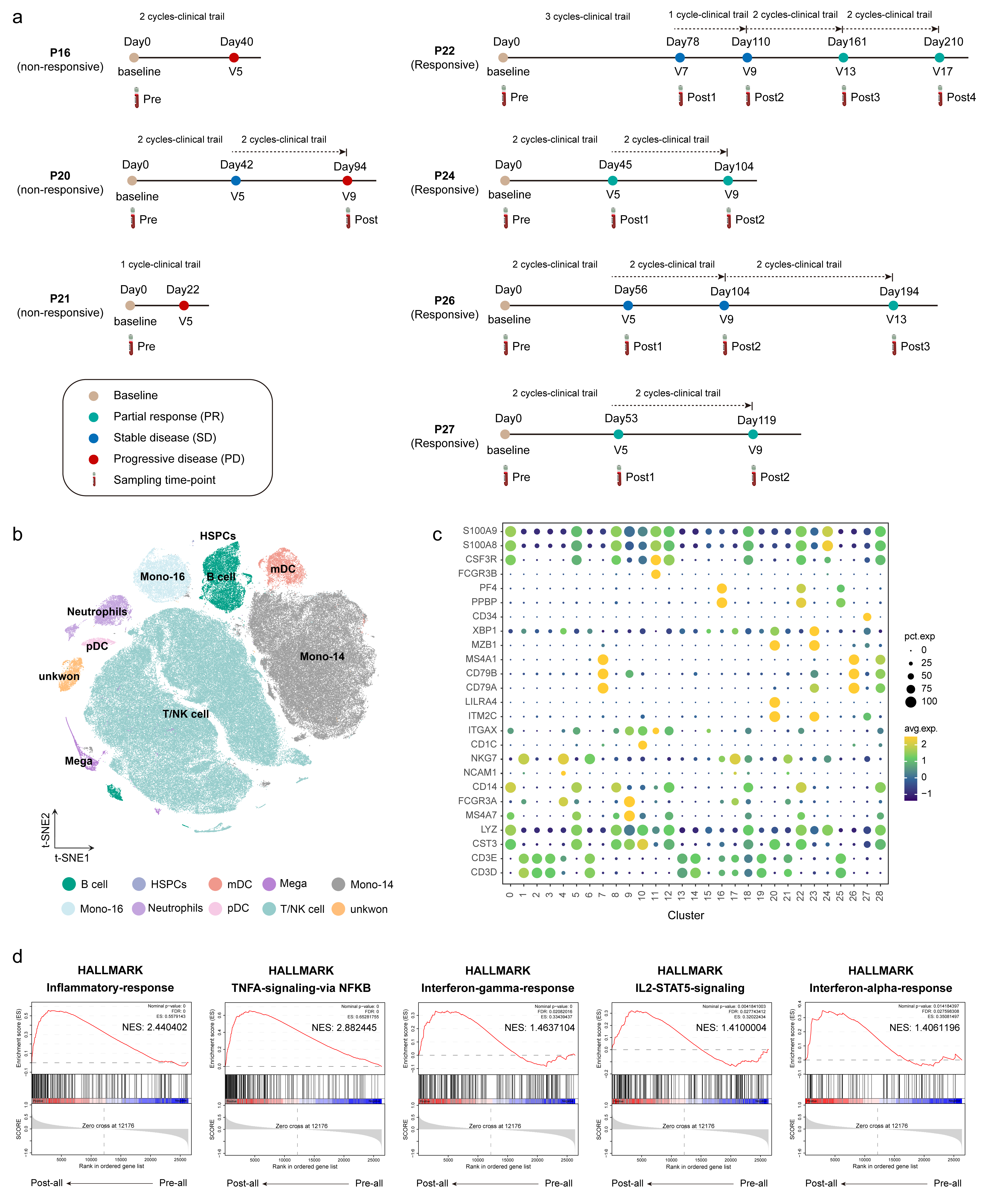


**Figure. S4. Single-cell transcriptional profiling of immune cells in response to the NK cell-based therapy. a** Overview of patients and scRNA-seq sampling timepoints across the clinical trial cycles. Patients with the best overall response (BOR) of complete response (CR) and partial response (PR) were classified as responders, patients with stable disease (SD) or progressive disease (PD) were classified as nonresponders. **b** t-SNE plot showing the annotation for immune cell types. **c** Dot plot showing the expression levels of marker genes in the unsupervised clusters. **d** Gene set enrichment analysis (GSEA) profiles, showing the five representatively significant GSEA hallmarks pathways enriched in all post-treatment samples (post-all) (*P*-value < 0.05, FDR < 0.25 and normalized enrichment score (NES) > 1).

Figure. S5.


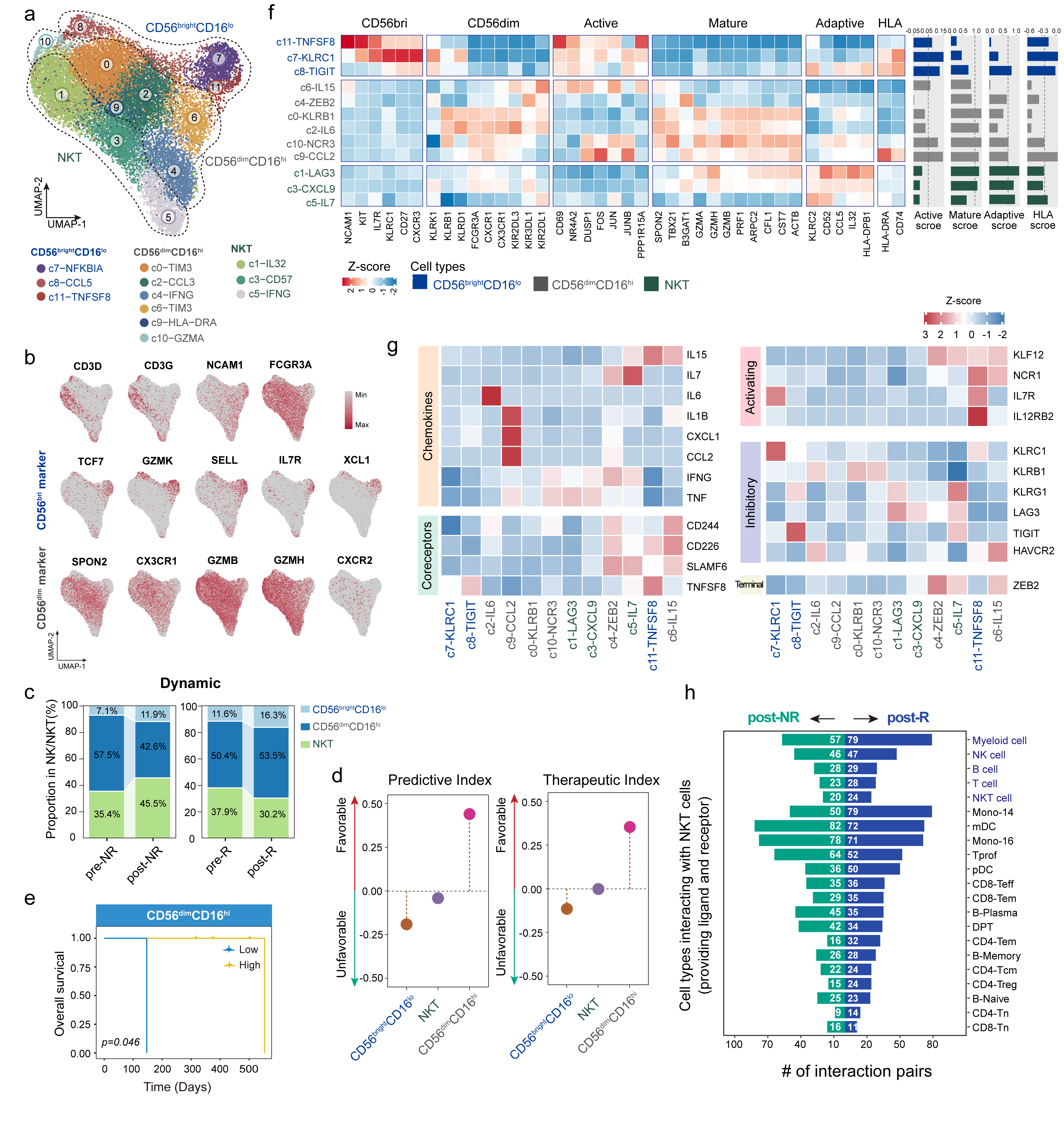


**Figure. S5. Construction of the circulating NK/NKT cells landscape in human pancreatic cancer. a** Uniform manifold approximation and projection (UMAP) plot visualization of CD56^bright^CD16^lo^ NK cells, CD56^dim^CD16^hi^ NK cells, and NKT cells derived from APC patients. Cluster annotations are indicated. **b** UMAP plots showing the signature genes of CD56^bright^CD16^lo^ NK cell, CD56^dim^CD16^hi^ NK cell, and NKT cells. **c** Bar chat showing the baseline proportion of annotated NK/NKT cells in responders (pre-R, n = 4) and nonresponders (pre-NR, n = 3). **d** The Pi and Ti analysis of NK/NKT cell annotated subtypes. **e** The Kaplan–Meier curve indicating the differences in OS between high- and low-proportion of post-treated CD56^dim^CD16^hi^ NK cells (n = 5, P20-post, P22-post3, P24-post1, P26-post3 and P27-post1)**.** *P*-value was determined by log-rank test. **f** Heatmap showing the expression pattern of functional genes for each cluster in NK/NKT cells (left panel), color is coded by the Z-score-scaled gene expression. Bar plots (right panel) represent the functional scores of each cluster. Each dashed line shows the median of the score. **g** Heatmap showing the selected functional gene sets expressed in each NK/NKT subset. Color is coded by the Z-score-scaled gene expression. **h** Bar chart showing the number of potential significant ligand-receptor pairs in NKT cells and other immune cells in post-NR (n = 1) and post-R (n = 4) samples. NKT cells providing ligand and receptors were calculated together.

Figure. S6.


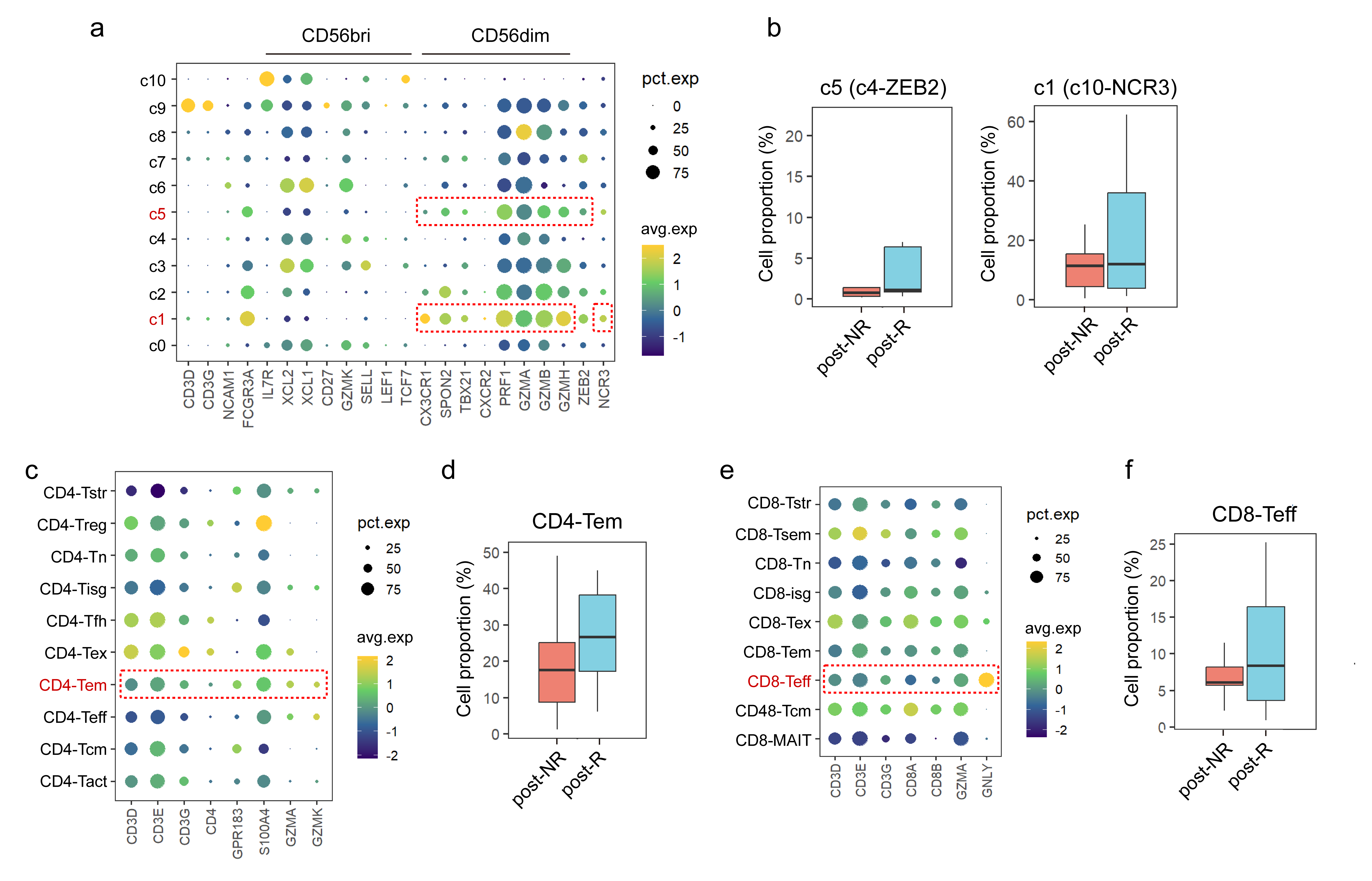


**Figure. S6. Characteristics and temporal dynamics of T/NK cell subsets in the public scRNA-seq dataset (GSE266919). a** Dot plot showing the expression pattern of functional genes and the signature genes of each NK cell cluster. The color scale represents the average gene expression level; dot size represents the percentage of cells expressing a given gene. **b** Boxplots showing the percentage of NK cell cluster c5 (left, representing c4-ZEB2 in our data) and c1 (right, representing c10-NCR3 in our data) post PD-L1 inhibitor treatment plus chemotherapy. post-R, post-treated samples of responders; post-NR, post-treated samples of nonresponders. **c** Dot plot showing the expression levels of marker genes in the CD4^+^ T cell subsets. CD4-Tn, CD4^+^ naive T cells; CD4-Tcm, CD4^+^ central memory T cells; CD4-Tact, CD4^+^ active T cells; CD4-Teff, CD4^+^ effector T cells; CD4-Tem, CD4^+^ effector memory T cells; CD4-Tisg, CD4^+^ IFN response related T cells; CD4-Tstr, CD4^+^ stressed T cells; Cd4-Tex, CD4^+^ exhausted T cells; CD4-Tfh, CD4^+^ follicular helper T cells; CD4-Treg, CD4^+^ regulatory T cells. **d** Boxplot showing the proportion of CD4-Tem following PD-L1 inhibitor treatment plus chemotherapy. **e** Dot plot showing the expression levels of marker genes in the CD8^+^ T cell subsets. CD8-Tn, CD8^+^ naive T cells; CD8-Tcm, CD8^+^ central memory T cells; CD8-Teff, CD8^+^ effector T cells; CD8-Tem, CD8^+^ effector memory T cells; CD8-Tisg, CD8^+^ IFN response related T cells; CD8-Tstr, CD8^+^ stressed T cells; Cd8-Tex, CD8^+^ exhausted T cells; CD8-Tfh, CD8^+^ follicular helper T cells; CD8-MAIT, CD8^+^ mucosal-associated invariant T cells. **f** Boxplot showing the percentage of CD8-Teff after PD-L1 inhibitor treatment plus chemotherapy.

Figure. S7.


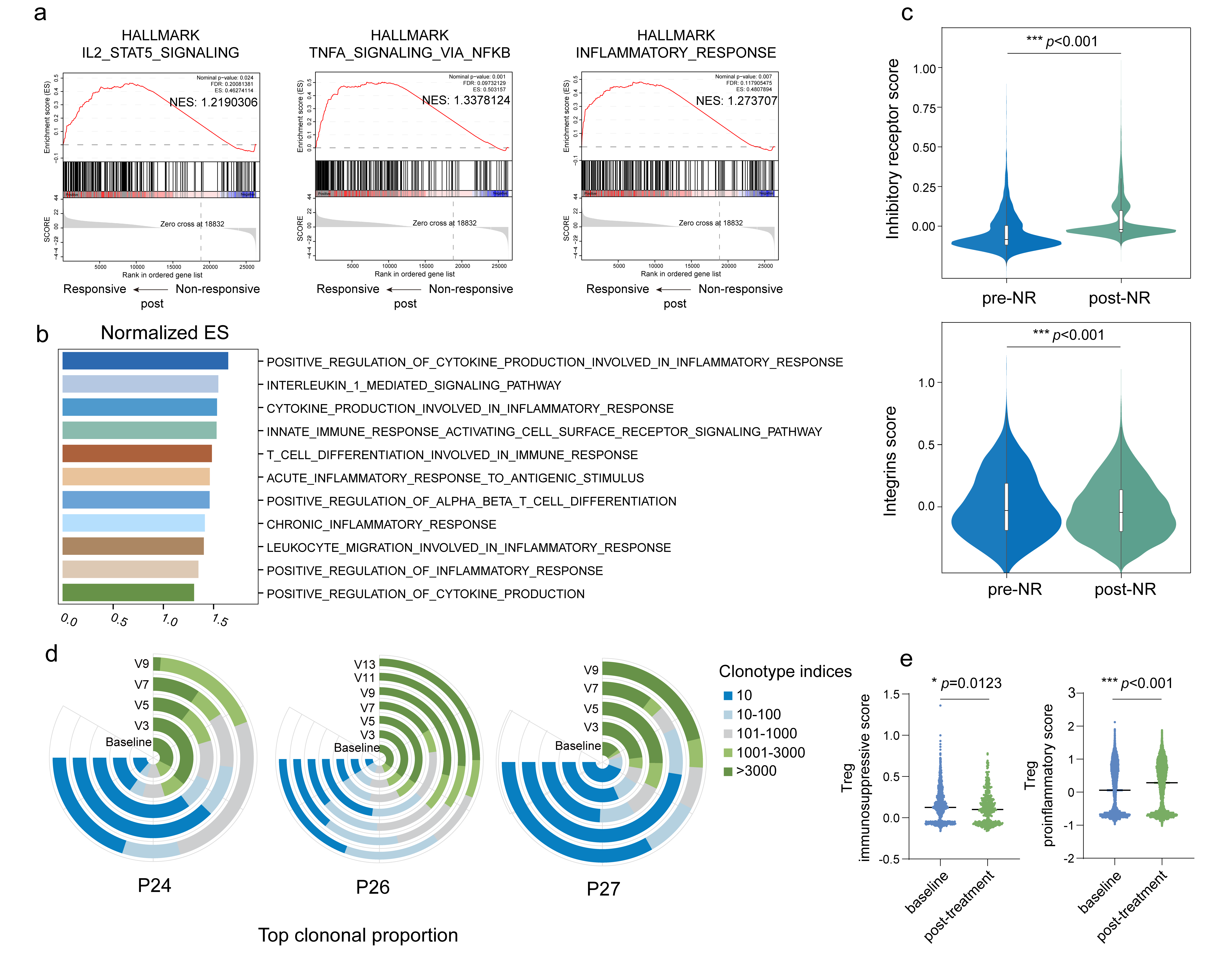


**Figure. S7. Characteristics and temporal dynamics of T cell subsets in human pancreatic cancer. a** GSEA analysis (Hallmark) showing the three representatively significant hallmark pathways enriched in post-treated responders (n=4), compared with nonresponder(n=1) (*P*-value < 0.05, FDR < 0.25 and NES > 1). **b** GSEA analysis (GO-BP) showing the 10 representatively significant GO BP-terms enriched in post-treated responders (n=4), compared with nonresponder(n=1) (*P*-value < 0.05, FDR < 0.25 and NES > 1). **c** Violin plots showing the dynamic alteration of inhibitory score and integrins score of T cells in nonresponder (n = 1). The *p* value was calculated by Wilcoxon test**. d** Distribution of TCRβ clonotypes frequency of top clonotype groups, sampled from the baseline to longitudinal visit timepoints across the clinical trial. **e** Comparison of Treg immunosuppressive score and Treg proinflammatory score of Treg cells between baseline samples (n = 7) and post-treated responders (n = 4, P22, P24, P26, and P27) (gene sets showed in Method). *P*-value < 0.05 considered statistic significant (**p* < 0.05, ***p* < 0.01, ****p* < 0.001).

| Table S1. Clinical characteristics of all patients (n = 25). | | | | | | | | | | | | |
| --- | --- | --- | --- | --- | --- | --- | --- | --- | --- | --- | --- | --- |
| Patient No. | Cohort | Age | Gender | Diagnosis | Disease status | Site of primary tumor | Number of metastatic sites | Degree of tumor differentiation | Dose Level (cells) | NK cells origin | NK cell administration (No. Cycles) | Clinical efficacy |
| P1 | 1 | 60 | Male | Pancreatic cancer | Metastatic | Head and neck | 1 | NA^#^ | 1 × 10^9^ | Peripheral blood | 3 | SD |
| P2 | 1 | 44 | Female | Pancreatic cancer | Metastatic | Head and neck | 2 | Poorly Differentiated | 1 × 10^9^ | Peripheral blood | 6 | PR |
| P3 | 1 | 62 | Male | Pancreatic cancer | Metastatic | Body and Tail | 1 | NA | 1 × 10^9^ | Peripheral blood | 4 | SD |
| P4 | 1 | 64 | Female | Pancreatic cancer | Metastatic | Body and Tail | 3 | Moderately Differentiated | 1 × 10^9^ | Peripheral blood | 5 | SD |
| P6 | 2 | 75 | Male | Pancreatic cancer | Metastatic | Body and Tail | 1 | Moderately Differentiated | 2 × 10^9^ | Cord blood | 3 | PR |
| P8 | 2 | 64 | Female | Pancreatic cancer | Locally advanced | Head and neck | 1 | NA | 2 × 10^9^ | Cord blood | 5 | SD |
| P9 | 2 | 67 | Female | Pancreatic cancer | Metastatic | Head and neck | 1 | NA | 2 × 10^9^ | Peripheral blood | 8 | SD |
| P10 | 2 | 67 | Male | Pancreatic cancer | Metastatic | Head and neck | 2 | NA | 2 × 10^9^ | Peripheral blood | 1 | NA |
| P11 | 2 | 67 | Male | Pancreatic cancer | Metastatic | Body and Tail | 1 | NA | 2 × 10^9^ | Peripheral blood | 4 | SD |
| P12 | 2 | 56 | Male | Pancreatic cancer | Metastatic | Head and neck* | 1 | Poorly Differentiated | 2 × 10^9^ | Cord blood | 4 | SD |
| P13 | 3 | 53 | Male | Pancreatic cancer | Metastatic | Body and Tail | 5 | Poorly Differentiated | 4 × 10^9^ | Peripheral blood | 2 | PD |
| P14 | 3 | 60 | Male | Pancreatic cancer | Metastatic | Body and Tail | 3 | Moderately Differentiated | 4 × 10^9^ | Peripheral blood | 1 | PD |
| P15 | 4 | 68 | Male | Pancreatic cancer | Metastatic | Body and Tail | 5 | NA | 8 × 10^9^ | Cord blood | 2 | SD |
| P16 | 4 | 68 | Male | Pancreatic cancer | Metastatic | Head and neck | 1 | NA | 8 × 10^9^ | Cord blood | 2 | PD |
| P17 | 3 | 56 | Male | Pancreatic cancer | Metastatic | Body and Tail | 3 | Moderately Differentiated | 4 × 10^9^ | Cord blood | 6 | SD |
| P18 | 4 | 48 | Female | Pancreatic cancer | Metastatic | Body and Tail | 2 | Poorly Differentiated | 8 × 10^9^ | Cord blood | 3 | SD |
| P19 | 3 | 51 | Male | Pancreatic cancer | Metastatic | Head and neck | 2 | NA | 4 × 10^9^ | Cord blood | 2 | NA |
| P20 | 3 | 65 | Male | Pancreatic cancer | Metastatic | Body and Tail | 4 | NA | 4 × 10^9^ | Cord blood | 4 | SD |
| P21 | 3 | 58 | Male | Pancreatic cancer | Metastatic | Body and Tail | 7 | NA | 4 × 10^9^ | Cord blood | 3 | PD |
| P22 | 4 | 69 | Male | Pancreatic cancer | Metastatic | Body and Tail | 1 | Poorly Differentiated | 8 × 10^9^ | Cord blood | 13 | PR |
| P23 | 4 | 61 | Male | Pancreatic cancer | Metastatic | Head and neck | 3 | Poorly Differentiated | 8 × 10^9^ | Cord blood | 2 | PD |
| P24 | 3 | 64 | Male | Pancreatic cancer | Locally advanced | Body and Tail | 0 | Moderately Differentiated | 4 × 10^9^ | Cord blood | 4 | PR |
| P25 | NA^#^ | 69 | Male | Pancreatic cancer | Metastatic | Head and neck | 2 | Moderately Differentiated | NA | NA | 0 | NA |
| P26 | 4 | 70 | Male | Pancreatic cancer | Locally advanced | Body and Tail | 0 | Moderately Differentiated | 8 × 10^9^ | Cord blood | 6 | PR |
| P27 | 4 | 71 | Male | Pancreatic cancer | Metastatic | Body and Tail | 1 | NA | 8 × 10^9^ | Cord blood | 4 | PR |
| *Tumoral site of P12 located both in neck, body and tail | | | | |  |  |  |  |  |  |  |  |
| #NA, not available | |  |  |  |  |  |  |  |  |  |  |  |

| **Table S2.** **Treatment-related adverse events (TRAEs) reported with NK-cell therapy in combination with the GS chemotherapy (n = 25).** | | | | |
| --- | --- | --- | --- | --- |
| **Preferred term**^a^**, n (%)** | | **Grade 1 or 2** | **Grade 3 or 4** | **Any** |
| Any AE | | 24 (96.0) | 14 (56.0) | 24 (96.0) |
| *Hematology* | | 22 (88.0) | 13 (52.0) | 22 (88.0) |
|  | Neutropenia | 9 (36.0) | 10 (40.0) | 19 (76.0) |
|  | Leukopenia | 13 (52.0) | 5 (20.0) | 18 (72.0) |
|  | Thrombocytopenia | 13 (52.0) | 3 (12.0) | 16 (64.0) |
|  | Anemia | 11 (44.0) | 1 (4.0) | 12 (48.0) |
|  | Aspartate aminotransferase increased | 9 (36.0) | 0 | 9 (36.0) |
|  | Alanine aminotransferase increased | 7 (28.0) | 2 (8.0) | 9 (36.0) |
|  | Alkaline phosphatase increased | 6 (24.0) | 1 (4.0) | 7 (28.0) |
|  | γ-glutamyltransferase increased | 4 (16.0) | 1 (4.0) | 5 (20.0) |
|  | Total bilirubin increased | 4 (16.0) | 0 | 4 (16.0) |
|  | Direct bilirubin increased | 3 (12.0) | 0 | 3 (12.0) |
|  | Elevated lactate dehydrogenase | 1 (4.0) | 0 | 1 (4.0) |
|  | Creatine kinase increased | 1 (4.0) | 0 | 1 (4.0) |
| *Gastrointestinal disorders* | | 14 (56.0) | 0 | 14 (56.0) |
|  | Diarrhea | 2 (8.0) | 0 | 2 (8.0) |
|  | Abdominal pain | 1 (4.0) | 0 | 1 (4.0) |
|  | Mucositis oral | 1 (4.0) | 0 | 1 (4.0) |
|  | Constipation | 2 (8.0) | 0 | 2 (8.0) |
|  | Nausea | 8 (32.0) | 0 | 8 (32.0) |
|  | Vomiting | 3 (12.0) | 0 | 3 (12.0) |
|  | Hiccups | 1 (4.0) | 0 | 1 (4.0) |
|  | Gastroesophageal reflux disease | 1 (4.0) | 0 | 1 (4.0) |
| *Others* | |  |  |  |
|  | Rash | 5 (20.0) | 1 (4.0) | 6 (24.0) |
|  | Allergic dermatitis | 1 (4.0) | 0 | 1 (4.0) |
|  | Alopecia | 1 (4.0) | 0 | 1 (4.0) |
|  | Skin hyperpigmentation | 3 (12.0) | 0 | 3 (12.0) |
|  | Fatigue | 4 (16.0) | 0 | 4 (16.0) |
|  | Fever | 6 (24.0) | 0 | 6 (24.0) |
|  | Insomnia | 1 (4.0) | 0 | 1 (4.0) |
|  | Chill | 2 (8.0) | 0 | 2 (8.0) |
| ^a^Medical Dictionary for Regulatory Activities version 23.1, graded according to CTCAE version 5.0. | | | | |

| **Table S3. Treatment-emergent adverse events (TEAEs) reported with NK cell therapy in combination with the GS chemotherapy (n = 25).** | | | | | | |
| --- | --- | --- | --- | --- | --- | --- |
| Preferred term^a^, n (%) | | All cohort (n = 25) | | | | |
|  |  | Grade | | | | |
|  |  | 1 | 2 | 3 | 4 | Any |
| *Hematology* | |  |  |  |  |  |
|  | Neutropenia | 4 (16.0) | 5 (20.0) | 8 (32.0) | 2 (8.0) | 19 (76.0) |
|  | Leukopenia | 4 (16.0) | 9 (36.0) | 5 (20.0) | 0 | 18 (72.0) |
|  | Thrombocytopenia | 5 (20.0) | 8 (32.0) | 3 (12.0) | 0 | 16 (64.0) |
|  | Anemia | 8 (32.0) | 3 (12.0) | 2 (8.0) | 1 (4.0) | 14 (56.0) |
|  | Aspartate aminotransferase increased | 6 (24.0) | 3 (12.0) | 0 | 0 | 9 (36.0) |
|  | Alanine aminotransferase increased | 6 (24.0) | 1 (4.0) | 2 (8.0) | 0 | 9 (36.0) |
|  | Alkaline phosphatase increased | 6 (24.0) | 1 (4.0) | 1 (4.0) | 0 | 8 (32.0) |
|  | γ-GGT increased | 3 (12.0) | 3 (12.0) | 2 (8.0) | 0 | 8 (32.0) |
|  | Direct bilirubin increased | 4 (16.0) | 0 | 0 | 0 | 4 (16.0) |
|  | Total bilirubin increased | 5 (20.0) | 0 | 0 | 0 | 5 (20.0) |
|  | Hypoproteinemia | 6 (24.0) | 0 | 0 | 0 | 6 (24.0) |
|  | Hypoalbuminemia | 5 (20.0) | 0 | 0 | 0 | 5 (20.0) |
|  | Elevated lactate dehydrogenase | 3 (12.0) | 0 | 0 | 0 | 3 (12.0) |
|  | Hyperuricemia | 2 (8.0) | 0 | 0 | 0 | 2 (8.0) |
|  | Creatine kinase increased | 2 (8.0) | 0 | 0 | 0 | 2 (8.0) |
|  | Hypokalemia | 5 (20.0) | 0 | 0 | 0 | 5 (20.0) |
|  | Hypocalcemia | 1 (4.0) | 0 | 0 | 0 | 1 (4.0) |
| *Gastrointestinal disorders* | |  |  |  |  |  |
|  | Diarrhea | 3 (12.0) | 0 | 0 | 0 | 3 (12.0) |
|  | Abdominal pain | 1 (4.0) | 0 | 0 | 0 | 1 (4.0) |
|  | Mucositis oral | 1 (4.0) | 0 | 0 | 0 | 1 (4.0) |
|  | Constipation | 5 (20.0) | 0 | 0 | 0 | 5 (20.0) |
|  | Nausea | 7 (28.0) | 1 (4.0) | 0 | 0 | 7 (28.0) |
|  | Vomiting | 2 (8.0) | 1 (4.0) | 0 | 0 | 3 (12.0) |
|  | Hiccups | 0 | 1 (4.0) | 0 | 0 | 1 (4.0) |
|  | Gastroesophageal reflux disease | 1 (4.0) | 0 | 0 | 0 | 1 (4.0) |
|  | Biliary tract infection | 0 | 0 | 1 (4.0) | 0 | 1 (4.0) |
|  | Gastrointestinal obstruction | 0 | 1 (4.0) | 0 | 0 | 1 (4.0) |
| *Cardiovascular disorders* | |  |  |  |  |  |
|  | Hypertension | 1 (4.0) | 0 | 0 | 0 | 1 (4.0) |
|  | Palpitations | 2 (8.0) | 0 | 0 | 0 | 2 (8.0) |
|  | Deep venous thrombosis | 0 | 2 (8.0) | 0 | 0 | 2 (8.0) |
| *Nervous system disorders* | |  |  |  |  |  |
|  | Anxiety | 1 (4.0) | 0 | 0 | 0 | 1 (4.0) |
|  | Dizziness | 1 (4.0) | 0 | 0 | 0 | 1 (4.0) |
|  | Insomnia | 5 (20.0) | 0 | 0 | 0 | 5 (20.0) |
|  | Memory impairment | 1 (4.0) | 0 | 0 | 0 | 1 (4.0) |
|  | Vision decreased | 1 (4.0) | 0 | 0 | 0 | 1 (4.0) |
| *Others* | |  |  |  |  |  |
|  | Dysuria | 1 (4.0) | 0 | 0 | 0 | 1 (4.0) |
|  | Ketonuria | 1 (4.0) | 0 | 0 | 0 | 1 (4.0) |
|  | Rash | 6 (24.0) | 1 (4.0) | 1 (4.0) | 0 | 8 (32.0) |
|  | Allergic dermatitis | 0 | 1 (4.0) | 0 | 0 | 1 (4.0) |
|  | Alopecia | 1 (4.0) | 0 | 0 | 0 | 1 (4.0) |
|  | Skin hyperpigmentation | 3 (12.0) | 0 | 0 | 0 | 3 (12.0) |
|  | Pain | 1 (4.0) | 0 | 0 | 0 | 1 (4.0) |
|  | Fatigue | 6 (24.0) | 0 | 0 | 0 | 6 (24.0) |
|  | Fever | 7 (28.0) | 1 (4.0) | 0 | 0 | 7 (28.0) |
|  | Chills | 1 (4.0) | 1 (4.0) | 0 | 0 | 2 (8.0) |
| ^a^Medical Dictionary for Regulatory Activities version 23.1, graded according to CTCAE version 5.0. | | | | | | |

| **Table S4. Efficacy outcomes of advanced pancreatic cancer assessed by cohorts at different dose levels (n=19).** | | | | | |
| --- | --- | --- | --- | --- | --- |
| Responses  (RECIST v1.1) | | Cohort 1 | Cohort 2 | Cohort 3 | Cohort 4 |
|  |  | (1 × 10^9^, n = 3) | (2 × 10^9^, n = 5) | (4 × 10^9^, n = 6) | (8 × 10^9^, n = 5) |
| Best overall response, | |  |  |  |  |
|  | PR, No. (%) | 1 (33.3) | 1 (20.0) | 1 (16.7) | 3 (60.0) |
|  | SD, No. (%) | 2 (66.7) | 4 (80.0) | 2 (33.3) | 0 |
|  | PD, No. (%) | 0 | 0 | 3 (50.0) | 2 (40.0) |
| ORR, No. (%) [95% CI] | | 1 (33.3) [0.8-90.6] | 1 (20.0) [0.5, 71.6] | 1 (16.7) [0.4, 64.1] | 3 (60.0) [14.7, 94.7] |
| DCR, No. (%) [95% CI] | | 3 (100) [29.2-100] | 5 (100) [47.8, 100] | 3 (50.0) [11.8, 88.2] | 3 (60.0) [14.7, 94.7] |
| Abbreviations: CR, complete response; DCR, disease control rate; NR, not reached. | | | | | |
| ORR, objective response rate; PD, progressive disease; PR, partial response; SD, stable disease. | | | | | |
|  | | | | | |

| **Table S5. The maximum baseline tumor long diameter of each scRNA-seq sample used for Pi analysis.** | | |
| --- | --- | --- |
| Patient No. | Sample name | Maximum long diameter (mm) |
| P16 | P16-pre | 33 |
| P20 | P20-pre | 27 |
| P21 | P21-pre | 81 |
| P22 | P22-pre | 64 |
| P24 | P24-pre | 42 |
| P26 | P26-pre | 65 |
| P27 | P27-pre | 18 |

| **Table S6. Shrinkage of tumor size for each scRNA-seq sample used for Ti analysis.** | |
| --- | --- |
| Patient No. | Shrinkage of tumor |
| P22 | 34.3% |
| P24 | 40.0% |
| P26 | 30.7% |
| P27 | 55.6% |

**References**

1. Zhang, Y. *et al.* Single-cell analyses reveal key immune cell subsets associated with response to PD-L1 blockade in triple-negative breast cancer. *Cancer Cell* **39**, 1578-1593.e1578 (2021).

2. Stephenson, E. *et al.* Single-cell multi-omics analysis of the immune response in COVID-19. *Nat. Med.* **27**, 904-916 (2021).

3. Zhang, J.-Y. *et al.* Single-cell landscape of immunological responses in patients with COVID-19. *Nat. Immunol.* **21**, 1107-1118 (2020).

4. Krämer, B. *et al.* Early IFN-α signatures and persistent dysfunction are distinguishing features of NK cells in severe COVID-19. *Immunity* **54**, 2650-2669.e2614 (2021).

5. Yang, C. *et al.* Heterogeneity of human bone marrow and blood natural killer cells defined by single-cell transcriptome. *Nat. Commun.* **10**, 3931 (2019).

6. Liu, B. *et al.* Temporal single-cell tracing reveals clonal revival and expansion of precursor exhausted T cells during anti-PD-1 therapy in lung cancer. *Nature Cancer* **3**, 108-121 (2022).

7. Yan, H. *et al.* Primary Tr1 cells from metastatic melanoma eliminate tumor-promoting macrophages through granzyme B- and perforin-dependent mechanisms. *Tumor Biol.* **39**, 1010428317697554 (2017).

8. Overacre-Delgoffe, A. E. & Vignali, D. A. A. Treg Fragility: A Prerequisite for Effective Antitumor Immunity? *Cancer Immunol. Res.* **6**, 882-887 (2018).

9. Li, Y. *et al.* Potential anti-tumor effects of regulatory T cells in the tumor microenvironment: a review. *J. Transl. Med.* **22**, 293 (2024).

10. Zhang, Y. *et al.* Distinct cellular mechanisms underlie chemotherapies and PD-L1 blockade combinations in triple-negative breast cancer. *Cancer Cell* **43**, 446-463.e447 (2025).
