# Supplementary material for: Dynamic transcriptional immune landscape in response to NK-cell therapy combined with gemcitabine plus S-1 in advanced pancreatic cancer: a phase 1b/2 trial
Source: Signal Transduct Target Ther. 2025 Nov 21;10:381. doi: 10.1038/s41392-025-02488-1 (PMC12635223; doi:10.1038/s41392-025-02488-1)
Supplement: Supplementary file 2 — Trial Protocol [file 41392_2025_2488_MOESM2_ESM.docx]

Title:

**A single-arm, single-center clinical trial of activated NK cells immunotherapy for advanced pancreatic cancer to evaluate the efficacy and safety of NK cell therapy combined with gemcitabine plus S-1**

**Study protocol**

Protocol version: Version 1.0

Version date: 2018.12.13

**Principal Investigator:**

*Jie Ma, M.D., Ph.D.*

Professor and Director at Center of Biotherapy

Tel:86-10-85133661

*Yunbo Zhao, M.D.*

Chief Physician at Department Oncology

Tel:86-10-85136716

**Research Institute:** Beijing Hospital

**Table of Contents**

**1. Background information………………………………………………………1**

1. **Trial Objectives………………………………………………………………….5**
   1. Primary outcomes………………………………………………………..…..5
   2. Secondary outcomes…………………………………………………………5

2.3 Exploratory outcomes………………………………………………………..5

1. **Trial Design……………………………..…………………………..……………5**

3.1. The inclusion and exclusion criteria……………………………….…….5

3.2. Interventions……………………………………………………...………..7

3.3. Infusion Requirements for NK Cells infusion…………………..……….8

3.4 Dose modification…………………………………………………………11

3.5. Safety evaluation…………………………………………………...…….11

3.6. Response evaluation………………………………………………...…..14

3.7. Follow-up………………………………………………………………….15

3.8. Statistical analysis…………………………………………..……………15

**4. Quality Control…………………………………………………………………15**

**5. Right to Refuse Participation or Withdraw from the Study……………16**

**6. Privacy and Confidentiality Issues…………………………………………16**

**1.Background information**

In recent years, cancer immunotherapy has rapidly evolved and is now recognized as the fourth pillar of cancer treatment, following surgery, radiochemotherapy, and targeted therapy. Since the first engineered immune cell therapy targeting human melanoma based on tumor-specific T-cell receptors (TCRs) was developed in 1994, various chimeric antigen receptor T (CAR-T) cell technologies carrying different tumor-specific antigens have been successively developed. The first CAR-T clinical trial began in 2008, and its application has since been expanded to multiple cancers, including chronic lymphocytic leukemia (CLL)^[1]^, acute lymphoblastic leukemia (ALL)^[2]^, non-Hodgkin lymphoma (NHL)^[2]^, acute myeloid leukemia (AML)^[3]^, renal cell carcinoma (RCC)^[4]^, colorectal cancer^[5]^, and ovarian cancer^[6]^. On August 31 and October 19, 2017, the US FDA approved the CAR-T therapies Kymriahand Yescarta for the treatment of ALL.

However, both CAR-T and TCR technologies have significant limitations and side effects. For example, the mismatch between the engineered TCR chains and the endogenous TCR chains can significantly reduce the expression of TCRs on the T cell surface, thereby diminishing their anti-tumor efficacy^[7,8].^ Moreover, CAR-T therapy can induce severe inflammatory side effects. Morgan reported a case of a patient with metastatic colorectal cancer who died of acute respiratory failure five days after infusion of anti-ERBB2 CAR-T cells, likely due to the recognition and lysis of normal lung cells expressing ERBB2 by CAR-T cells, resulting in the secretion of inflammatory cytokines such as TNF-α and IFN-γ, and subsequent pulmonary toxicity and multi-organ dysfunction syndrome^[9]^.

With the significant advancements in cancer immunotherapy in recent years, the potential of natural killer (NK) cells in cancer immunotherapy has garnered considerable attention. As part of the innate immune system, NK cells play a crucial role in immune regulation, tumor defense, and infection resistance, serving as the first line of defense against pathogenic diseases, including malignant tumors. Unlike T cells and B cells, NK cells are a group of innate killer cells with unique abnormal cell recognition capabilities, lacking major histocompatibility complex (MHC) and antibody production. They can kill cells lacking MHC class I without prior activation^[10]^, and possess immune clearance and immune surveillance functions. Therefore, NK cells play an important role in resisting tumors, particularly in the clearance of metastatic and minimal residual tumor cells^[11]^. The direct and rapid cytotoxic dissolution effect of NK cells, their broad targets range, and their ability to infiltrate solid tissues make them promising candidates for cancer cell therapy. Given the potential sever adverse events associated with many gene-engineered immune cells like CAR-T, such as "cytokine storms," and the high treatment costs, their clinical application is significantly restricted. However, the anti-tumor activities of NK cells are relatively well understood, and their clinical side effects are milder. Developing therapeutic NK cells for clinical use to meet the urgent needs of cancer patients is of great significance and imperative.

However, there are currently no mature technical standards or guidelines internationally, and the existing evaluation criteria are not fully applicable. Therefore, researching and establishing clinical evaluation standards suitable for these innovative NK cell products are crucial for promoting the leading position of China's pharmaceutical industry globally.

Pancreatic cancer, one of the most aggressive malignancies, has seen limited improvement in patient survival despite advances in conventional therapies. This has driven the exploration of innovative treatment modalities, with immunotherapy emerging as a promising avenue. Recent advances have highlighted the potential of NK cell therapy as a novel treatment modality for other solid tumors^[12]^. However, in pancreatic cancer, previous study were mainly preclinical or ongoing clinical trials, the clinical outcomes of NK cell therapy remain sparse, and the clinical evidence regarding the application of NK cells therapy in combination with gemcitabine plus S-1 (GS) have not been reported. Besides, there are few systematical studies on whether immunotherapy, especially NK cell therapy, can enhance the immune status.

Single-cell RNA sequencing (scRNA-seq) has revolutionized our understanding of the immune landscape of cancers^[13,14]^. This technology allows for the detailed characterization of immune cell populations within the tumor microenvironment (TME) or peripheral blood, revealing heterogeneity and functional states that were previously obscured by bulk analysis. This highlights the potential of modulating specific immune cell populations to enhance the immunotherapeutic efficacy. The integration of NK cell therapy and single-cell sequencing analysis could offer a promising approach to overcoming the challenges posed by pancreatic cancer. By leveraging the unique properties of NK cells and the detailed insights provided by single-cell sequencing, we can develop more targeted and effective immunotherapeutic strategies. This combination provides a framework for personalized treatment plans based on the specific immune landscape of individual tumors.

Here, we would initiate a first-in-human, single-arm, non-randomized, phase 1b/2 clinical trial (ChiCTR1900021764) in advanced pancreatic cancer patients with GS chemotherapy, to evaluate the safety and feasibility of allogeneic NK cells, as well as the effect on patients' immune system function in single-cell insight, to provide references for large-scale clinical studies, and to explore the optimal dose of NK cell therapy through dose increment studies.

**Reference**

1. M. Kalos, B. L. Levine, D. L. Porter et al., “T cells with chimeric antigen receptors have potent antitumor effects and can establish memory in patients with advanced leukemia,” Science Translational Medicine, vol. 3, no. 95, Article ID95ra73, 2011.
2. S. A. Grupp, M. Kalos, D. Barrett et al., “Chimeric antigen receptor-modified T cells for acute lymphoid leukemia,” The New England Journal of Medicine, vol. 368, no. 16, 2013pp. 1509–1518.
3. D. S. Ritchie, P. J.Neeson, A.Khot et al., “Persistence and efficacy of second-generation CAR T cell against the LeY antigen in acute myeloid leukemia,” Molecular Therapy, 2013.
4. C. H. J. Lamers, R. Willemsen, P. van Elzakker et al., “Immune responses to transgene and retroviral vector in patients treated with ex vivo-engineered T cells,” Blood, vol. 117, no. 1, pp. 72–82, 2011.
5. Q. Ma, R. M. Gonzalo-Daganzo, and R. P. Junghans, “Genetically engineered T cells as adoptive immunotherapy of cancer,” Cancer Chemotherapy and Biological Response Modifiers, vol. 20,pp. 315–341, 2002.
6. M. H. Kershaw, J. A. Westwood, L. L. Parker et al., “A phase I study on adoptive immunotherapy using gene-modified T cells for ovarian cancer,” Clinical Cancer Research, vol. 12, no. 20, pp.6106–6115, 2006.
7. S. Okamoto, J. Mineno, H. Ikeda et al., “Improved expression and reactivity of transduced tumor-specific TCRs in human lymphocytes by specific silencing of endogenous TCR,” Cancer Research, vol. 69, no. 23, pp. 9003–9011, 2009.
8. E. Provasi, P. Genovese, A. Lombardo et al., “Editing T cell specificity towards leukemia by zinc finger nucleases and lentiviral gene transfer,” Nature Medicine, vol. 18, no. 5, pp. 807–815, 2012.
9. R. A. Morgan, J. C. Yang, M. Kitano, M. E. Dudley, C. M. Laurencot, and S. A. Rosenberg, “Case report of a serious adverse event following the administration of t cells transduced with a chimeric antigen receptor recognizing ERBB2,” Molecular Therapy, vol. 18, no. 4, pp. 843–851, 2010.
10. Lanier LL. A renaissance for the tumour immunosurveillance hypothesis. Nat [7] Med 2001;7:1178–80.
11. Ljunggren HG, Karre K. In search of the 'missing self': MHC molecules and NK cell recognition[J]. Immunol Today, 1990, 11(7):237-244.
12. Handgretinger R, Lang P, André MC. Exploitation of natural killer cells for the treatment of acute leukemia. Blood 127, 3341-3349 (2016).
13. Zheng C, Zheng L, Yoo JK et al., Landscape of Infiltrating T Cells in Liver Cancer Revealed by Single-Cell Sequencing. Cell. 2017 Jun 15;169(7):1342-1356.e16. doi: 10.1016/j.cell.2017.05.035.
14. Guo X, Zhang Y, Zheng L et al., Global characterization of T cells in non-small-cell lung cancer by single-cell sequencing. Nat Med. 2018 Jul;24(7):978-985. doi: 10.1038/s41591-018-0045-3. Epub 2018 Jun 25. Erratum in: Nat Med. 2018 Oct;24(10):1628. doi: 10.1038/s41591-018-0167-7.

**2. Trial Objectives**

The aim of this study is to evaluate the safety and efficacy of allogeneic NK cell therapy in advanced pancreatic cancer (APC) patients, as well as comprehensively understanding the peripheral immune landscape in these patients with distinct clinical outcomes in single cell insight.

**2.1 Primary outcomes**

The primary endpoint was the safety and tolerability of the NK cell therapy combined with gemcitabine plus S-1 (GS) in patients with advanced pancreatic cancer.

**2.2 Secondary outcomes**

The second endpoints were response rate and survival, including the objective response rate (ORR), disease control rate (DCR), progress free survival and overall survival (PFS) and overall survival (OS).

**2.3 Exploratory outcomes**

The exploration end point was to estimate the underling dynamic peripheral immune landscape in response to this NK cell-based therapy for advanced pancreatic cancer patients.

**3. Trial Design**

**3.1. The inclusion and exclusion criteria**

**3.1.1 Inclusion criteria**

1. Age ≥ 18 and ≤ 75 years, male or female.
2. Histologically or imaging examination (CT or MRI) confirmed advanced pancreatic cancer.
3. Planned with the GS standard chemotherapy.
4. At least one measurable lesion according to the response evaluation criteria in solid tumors (RECIST) V1.1.
5. Karnofsky performance score ≥ 80.
6. Expected survival period exceeded 3 months.
7. Absolute neutrophil count ≥ 1.5 × 10^9^/L, platelet count ≥ 80 × 10^9^/L and hemoglobin ≥ 9 g/dL.
8. Total bilirubin ≤ 1.5-times upper limit of normal (ULN), alkaline phosphatase ≤ 5-times ULN, and aspartate aminotransferase (AST) and alanine aminotransferase (ALT) ≤ 3-times ULN (AST and ALT ≤ 5-times ULN for patients with liver metastasis).
9. Serum creatinine ≤ 1.5-times ULN or calculated creatinine clearance ≥ 50 mL/min.
10. Basically normal electrocardiogram, cardiac ejection fraction > 50% and no unhealed trauma.
11. Human immunodeficiency virus (HIV), hepatitis B virus (HBV), and hepatitis C virus (HCV) tested negative.
12. Signed informed consent.

**3.1.2 Exclusion criteria**

1. Known allergy to biologics or gemcitabine and S-1 chemotherapy agents.
2. Existing severe uncontrolled acute infection, purulent or chronic infection, and prolonged wound healing.
3. Severe heart disease, including: congestive heart failure, uncontrolled cardiac arrhythmias, unstable angina, myocardial infarction, severe valve disease, and resistant hypertension.
4. Severe neurologic or psychiatric, or mental disorders, poor adherence, and inability to cooperate and describe treatment responses.
5. Primary brain tumor or uncontrolled central nervous metastases, with obvious intracranial hypertension or neuropsychiatric symptoms.
6. Bleeding tendency.
7. Drug addicts.
8. History of solid organ transplant or hematopoietic stem cell transplant.
9. Suffered from any other immunological disease, long-term use of corticosteroids or other immunosuppressants, or treated with immunosuppressive drugs within 3 months prior to participation, and treated with glucocorticoids within the week prior to participation.
10. Women of childbearing potential must have agreed to practice effective contraception during the study and for 6 months after their last dose of study treatment (such as an intrauterine device, birth control pills, or condoms), a negative serum or urinary pregnancy test within 14 days prior to enrollment, and be non-lactating.
11. Other situations evaluated by the investigators were not suitable for the clinical trial.

**3.2. Interventions**

Each 21-day treatment cycle consisted of standard GS chemotherapy combined with NK cell administration during the inpatient care by physicians.

**3.2.1 Chemotherapy protocols**

In each cycle, patients received gemcitabine on days 1 and 8 (1,000 mg/m^2^ intravenously over 30 minutes), along with oral S-1 twice daily from day 1 to day 14. The daily S-1 dose was determined by body surface area: 60 mg for < 1.25 m², 80 mg for 1.25 to 1.5 m², 100 mg for ＞ 1.5 m². Patients were scheduled to receive 6-8 cycles of GS chemotherapy followed by S-1 monotherapy maintenance. Local treatments were permitted throughout.

**3.2.2 NK cell administration**

According to the design of the dose-escalation test, individuals were assigned into four dose-level cohorts: 1 × 10^9^ NK cells as an initial dose, 2 × 10^9^ NK cells, 4 × 10^9^ NK cells and 8 × 10^9^ NK cells. The expanded NK cell products were infused intravenously between days 14 and 21 following chemotherapy, aiming to minimize the potential hematological toxicity to the infused NK cells. NK cells were administered once per cycle in the lower-dose cohorts (1 × 10^9^ cells, 2 × 10^9^ cells, and 4 × 10^9^ cells), while the highest-dose cohort (8 × 10^9^ cells) received half the total dose fractionated over two consecutive days to mitigate risks associated with large-volume infusion. NK cell infusion was combined with the first four cycles of GS chemotherapy, unless unacceptable adverse effects, disease progression, or patient withdrawal occurred. Patients who expressed a strong desire to continue NK cell therapy beyond four cycles could receive compassionate treatments, with the treatment cycle frequency determined by the investigators based on individual tolerance and response.

**3.2.3 Visit arrangement**

The screening period lasted no longer than 28 days. After signing the informed consent, advanced pancreatic cancer medical history, treatment history, baseline characteristics were collected, and relevant examinations were performed to determine whether they were suitable for this study.

The treatment period: A 21-day treatment cycle included standard GS chemotherapy and NK cell administration during inpatient care by the physicians. CT scanning or MRI were performed at baseline and every two-cycles treatment thereafter until disease progression. Two visits would be arranged per cycle (before gemcitabine administration and NK cell infusion, respectively). Peripheral blood samples will be drawn every cycle (for bulk TCR Vβ repertoire sequencing) or every two cycles of the trial (for single-cell RNA sequencing).

**3.3. Infusion Requirements for NK Cells infusion**

**3.3.1 NK cells manufacturing and release criteria:**

Peripheral blood (PB) was obtained from direct relatives of patients, and cord blood (CB) was collected from fetuses at delivery at Beijing Hospital. Mononuclear cells were isolated via density gradient centrifugation via Lymphocyte Separation Medium (Tianjin Haoyang Biological Manufacture Co., Ltd. Tianjin, China) under GMP-compliant conditions. NK cells were activated and expanded over approximately two weeks according to the manufacturer’s protocol for an *ex vivo* NK Cell Expansion Kit (Beijing Wukang Xinxing Technology Co., Ltd., Beijing, China), with media containing heat-inactivated autologous plasma and recombinant human interleukin-2 (Beijing SL Pharmaceutical Co., Ltd., Beijing, China). The cells were harvested on days 14 to 16, washed, and resuspended in 100 mL of saline-based solution containing 0.6% human serum albumin (albumin, Baxalta Pharmaceutical Company, USA) for immediate intravenous administration to patients.

Quality control included sterility testing via the BACTEC™ Blood Culture System (BD Diagnostics, Sparks, MD, USA), mycoplasma detection via quantitative real-time PCR-based kits (TransGen Biotech, Beijing, China), endotoxin assessment via the LAL assay (Xiamen Bioendo Technology Co., LTD, Xiamen, China), and viability measurement via ViaStain™ AOPI staining (Revvity, Waltham, MA, USA). The purity of CD3⁻CD56⁺ NK cells was determined via flow cytometry (BD FACSCanto II). The following release criteria were used: sterility and mycoplasma negativity, endotoxin concentration < 0.25 EU/mL, viability ≥ 85%, and CD3^-^CD56^+^ NK cell purity ≥ 70%. Cytotoxic activity was evaluated against the K-562 cell lines via a time-resolved fluorescence-based cytotoxicity assay (DELFIA®; Revvity). Specific lysis was calculated after coculture at various E:T ratios. Products that achieved ≥ 40% specific lysis of K-562 cells at an E:T ratio of 2.5:1 were released from the infusion.

**3.3.2 Infusion Environment:**

The NK cell injection is administered on the final day of the patient's hospitalization, within the ward. Temperature and humidity control should follow the routine requirements of the hospital's nursing department.

**3.3.3 Reception of NK Cells products:**

Upon arrival at the hospital under cold condition, the NK cells products should be used immediately on the same day. A special person is responsible for receiving the NK cell products and registering it in the NK cell products registration form to describe the situation of the NK cells at the time of receipt. NK cell injection should be handled gently, not violently shaken, not squeezed.

**3.3.4 Infusion Preparation:**

The intravenous infusion of the NK cells is to be performed by a designated individual who has undergone specific training. The designated individual must wash their hands, change into work attire, and prepare the necessary items for infusion, including a treatment cart, a kidney dish, a disposable sterilization pack, an infusion set, a No. 7 scalp needle, 0.9% saline solution, scissors, infusion labels, and hand sanitizer.

**3.3.5 Infusion Procedure:**

Thoroughly inspect the cell suspension for any flocculent material or turbidity. Verify that the patient information on the infusion label matches that on the cell label. Prepare all necessary items and transport the treatment cart to the patient's bedside. Wash hands again with hand sanitizer and wear a mask. Explain to the patient that the cell infusion is about to begin and ensure they are prepared. Remove the infusion set from its packaging using scissors. Insert the large needle into the medication vial. Hold the Murphy's drip tube upside down, hang it on the infusion stand, and expel all air. Close the regulator and check for any air bubbles in the infusion tubing. Select a peripheral vein in the forearm or elbow that is thick, straight, and elastic to ensure the required infusion rate for NK cells. Instruct the patient to assume a comfortable position.

Place a disposable treatment towel under the selected vein. Apply a tourniquet 5-6 cm above the puncture site. Perform routine skin disinfection with a diameter of at least 5 cm and allow it to dry. Prepare for venipuncture. Before the infusion of cells, gently reverse the infusion bag 3-4 times up and down to fully mix, and then infuse intravenously according to the routine infusion procedures, strictly in accordance with the principle of aseptic operation. NK cells are flushed with 0.9% normal saline before and after infusion so that all cells could be injected into the patient. Due to the large NK cell particles, the drip rate is controlled at about 15 drops/min in the first 10-15 minutes, and the drip rate is adjusted at about 40-60 drops/min after 15 minutes if there is no discomfort reaction. The patient's vital signs and general condition are closely observed by medical staff during the whole process of cell transfusion. If the patient experienced discomfort, the drip rate need slow down If symptoms persist, pause the infusion and provide symptomatic treatment. At the same time, Monitor the patient's temperature before and after the infusion. After needle removal, instruct the patient to apply pressure to the puncture site for at least 5 minutes. Observe the patient for one hour after the infusion and measure vital signs at the end of the one-hour observation period.

**3.4 Dose modification**

The adverse reactions to the chemotherapy (gemcitabine plus S-1) would be managed by the investigator in accordance with the instructions.

For adverse reactions related to NK cells, The investigators identify the adverse events according to the National Cancer Institute's Common Terminology Criteria for Adverse Events (CTCAE V4.03), and they will use their clinical experience to distinguish whether the adverse reactions are caused by conventional chemotherapeutic agents or by NK cells. If the investigators determine that the adverse reaction is caused by NK cells, the following protocol will be followed: If dose-limiting toxicity occurs in 1 of the 3 cases in group 1, the enrollment of 3 patients in the next group may continue. If dose-limiting toxicity occurs in 1 more of the 3 patients in the next group, i.e., in 2 or more of the 6 patients, the amount of NK cells used should be halved to 0.5 x 10^9^. If no DLT occurs at the current dose of 2 x 10^9^, the dose of NK cells will be escalated to 4 x 10^9^ units, with no dose-limiting toxicity, and the NK cell dose continues to be increased to 8 x 10^9^ units.

**3.5. Safety evaluation**

Safety assessment will include monitoring and recording all adverse events (AEs), including serious adverse events (SAEs), laboratory tests (liver and kidney function, complete blood count, urinalysis, stool examination), electrocardiograms (ECGs), vital signs, and physical examinations.

**3.5.1 Definition of Adverse Events**

An adverse event (AE) is defined as any untoward medical occurrence in a clinical trial that appears after the patient has signed the informed consent form. However, it does not necessarily have a causal relationship with the treatment. It includes any new event or any worsening in severity and frequency compared to the baseline condition, including abnormal results from diagnostic methods such as laboratory tests and physical examinations.

**3.5.2 Collection and Recording of Adverse Events**

Investigators are responsible for collecting and recording all adverse events for each patient from the time of signing the informed consent form until the end of the extended study period. Investigators are responsible for observing, recording, and following up on all adverse events that occur during the study process, regardless of their cause.

Any adverse event that occurs during the trial, regardless of its severity or whether it is related to the investigational drug, must be recorded in the adverse event section of the electronic case report form. This includes the date of occurrence, symptoms, severity (CTCAE V5.0), duration, management measures (continuation of medication, dose reduction, temporary discontinuation followed by resumption, permanent discontinuation, and others), and outcome (resolution with sequelae, resolution without sequelae, persistence, death, and others). Investigators must also evaluate the relationship with the investigational drug, considering comorbidities and concomitant medications/non-pharmacological treatments, and sign and date the records. Adverse events should be recorded using medical terminology, and a diagnosis of the disease should be provided rather than simply listing symptoms and signs.

Investigators must determine whether abnormal laboratory test results are clinically significant. If an abnormal laboratory value is clinically significant, it should be judged whether it is an adverse event, and the judgment should be recorded in the source document and accordingly recorded in the electronic case report form. If unexplained abnormal laboratory values occur, they must be retested or followed up until the values return to the normal range or baseline level and/or a reasonable and sufficient explanation is provided and recorded in the source document and electronic case report form.

Investigators should conduct clinical follow-up for patients experiencing adverse events until the adverse event resolves (returns to normal or baseline status), stabilizes, or has a reasonable explanation. Disease progression recorded according to the RECIST V1.1 (including death due to disease progression) should not be reported as an AE or SAE. Deaths, hospitalizations/extended hospital stays, or disabilities caused by the tumor itself that meet the criteria for SAEs should not be reported as SAEs. However, direct complications of the tumor that meet the SAE criteria must be reported as SAEs.

**3.5.3 The criteria for assessing dose-limiting toxicity**

*Hematological:*

1. Grade 4 neutropenia lasting for more than 7 days.
2. Febrile neutropenia.
3. Neutropenic infection of Grade 3 or higher.
4. Thrombocytopenia with bleeding of Grade 3 or higher.
5. Grade 4 thrombocytopenia.
6. Grade 4 anemia.

*Non-hematological:*

1. Grade 3 or higher nausea, vomiting, and diarrhea despite optimal supportive care.
2. Any other clinically significant non-hematological toxicity of Grade 3 or higher (excluding asymptomatic biochemical abnormalities without clinical significance that resolve to Grade 2 or lower within 7 days).
3. Any grade of dose-limiting toxicity as determined by the investigator.

**3.6. Response evaluation**

If a patient has undergone MRI or contrast-enhanced CT scans within 28 days prior to screening, and if these scans are deemed acceptable for the clinical study by the investigator, the results of these MRI or contrast-enhanced CT scans may be used for the screening evaluation. All patients participating in this study are required to undergo contrast-enhanced CT scans or MRI during the screening period, and every two cycles of treatment. All scan images obtained from the three time points must be analyzed using the same technique and evaluated for efficacy by designated researchers.

Contrast-enhanced CT scans must meet the imaging standards for lesions in each organ. Tumor response will be evaluated according to the RECIST V1.1 criteria. For spiral CT, the minimum size of target lesions should be ≥ 10 mm, while for conventional CT, the minimum size should be ≥ 20 mm. In this study, plain X-rays, ultrasound, and physical examination are not accepted as methods for monitoring measurable target lesions.

**3.7. Follow-up**

Patients will be followed up every 3 months for two years after the end of treatment.

**3.8. Statistical analysis**

In the initial phase of this study, we plan to recruit 30 participants diagnosed with advanced pancreatic cancer. Sample size estimation for this exploratory single-arm trial was determined according to the requirements of the project plan. After the clinical trial protocol is finalized, the statistical analysis plan will be developed by the statistical staff. If any changes occur in the statistical analysis, they will be documented in accordance with the operational procedures of the Clinical Research Steering Committee, with both the planned and additional statistical results clearly reported in the summary report. SAS 9.4 statistical software will be used.

Descriptive statistical methods will be employed for the analysis of baseline data from enrolled subjects. This will include a descriptive analysis of the actual number of patients enrolled, the number of dropouts and excluded cases, as well as demographic and other baseline characteristics. For quantitative indicators, descriptive statistics will be calculated, including mean, standard deviation, median, minimum, maximum, lower quartile (Q1), and upper quartile (Q3). For categorical indicators, the number and percentage of cases in each category will be reported.

**4. Quality Control**

Both the sponsor and the investigators are required to fulfill their respective responsibilities and strictly adhere to the clinical trial protocol, employing standard operating procedures (SOPs) to ensure the implementation of a robust quality control and quality assurance system for the clinical trial.

Clinical researchers and monitors should be trained in Good Clinical Practice (GCP) and possess relevant experience in clinical trial monitoring. Their role is to safeguard the rights and welfare of patients participating in the clinical trial, ensure the authenticity, accuracy, and completeness of trial data records and reports, and verify that the trial is conducted in accordance with the approved protocol, the Good Clinical Practice guidelines, and relevant regulations. The frequency of monitor visits should be sufficient to meet the needs of quality control for the clinical trial. Following each visit, the monitor shall report the findings to the principal investigators.

**5. Right to Refuse Participation or Withdraw from the Study**

The decision of a participant to not join or to discontinue involvement in this study will in no way affect their rights. Additionally, participants retain the right to withdraw from the study at any point in time. The researchers of this study reserve the right to determine whether participants are eligible for the study based on the specified inclusion criteria.

**6. Privacy and Confidentiality Issues**

All medical and personal information obtained from participants will be rigorously safeguarded. During the course of the study, identifying details such as names and gender will be replaced with unique codes or numbers to ensure anonymity. This anonymized data will be the only information utilized in the study, and it will be handled with the strictest confidentiality. When findings are disseminated through academic publications, no personally identifiable information will be disclosed. This measure ensures that participants' identities remain fully protected. All records pertaining to participants' involvement in the study, as well as any associated clinical and administrative documentation, will be securely maintained by Beijing Hospital. Access to these records will be strictly restricted to authorized personnel only, and unauthorized access is explicitly prohibited.
